# CD38 restrains the activity of extracellular cGAMP in a model of multiple myeloma

**DOI:** 10.1016/j.isci.2024.109814

**Published:** 2024-04-25

**Authors:** Lorenzo Cuollo, Samuele Di Cristofano, Annamaria Sandomenico, Emanuela Iaccarino, Angela Oliver, Alessandra Zingoni, Marco Cippitelli, Cinzia Fionda, Sara Petillo, Andrea Kosta, Valentina Tassinari, Maria Teresa Petrucci, Francesca Fazio, Menotti Ruvo, Angela Santoni, Domenico Raimondo, Alessandra Soriani

**Affiliations:** 1 Department of Molecular Medicine, Sapienza University of Rome, Laboratory affiliated to Istituto Pasteur Italia – Fondazione Cenci Bolognetti, Rome, Italy; 2 Institute of Biostructures and Bioimaging, CNR, Naples, Italy; 3 University of Campania “Luigi Vanvitelli”, Caserta, Italy; 4 Hematology, Department of Translational and Precision Medicine Azienda Policlinico Umberto I, Sapienza-Rome, Italy; 5 IRCCS Neuromed, Pozzilli, Italy

**Keywords:** Biochemical mechanism, Molecular biology, Components of the immune system, Cell biology, Cancer, In silico biology

## Abstract

2′3′-cyclic guanosine monophosphate–adenosine monophosphate (cGAMP) is the endogenous agonist of STING; as such, cGAMP has powerful immunostimulatory activity, due to its capacity to stimulate type I interferon-mediated immunity. Recent evidence indicates that cancer cells, under certain conditions, can release cGAMP extracellularly, a phenomenon currently considered important for therapeutic responses and tumor rejection. Nonetheless, the mechanisms that regulate cGAMP activity in the extracellular environment are still largely unexplored.

In this work, we collected evidence demonstrating that CD38 glycohydrolase can inhibit extracellular cGAMP activity through its direct binding.

We firstly used different cell lines and clinical samples to demonstrate a link between CD38 and extracellular cGAMP activity; we then performed extensive *in silico* molecular modeling and cell-free biochemical assays to show a direct interaction between the catalytic pocket of CD38 and cGAMP. Altogether, our findings expand the current knowledge about the regulation of cGAMP activity.

## Highlights

• Doxorubicin increases the release of extracellular cGAMP by myeloma cells
• CD38 inhibition causes the accumulation of extracellular cGAMP
• CD38 inhibits extracellular cGAMP activity through its direct binding

## Graphical abstract

**Figure:**
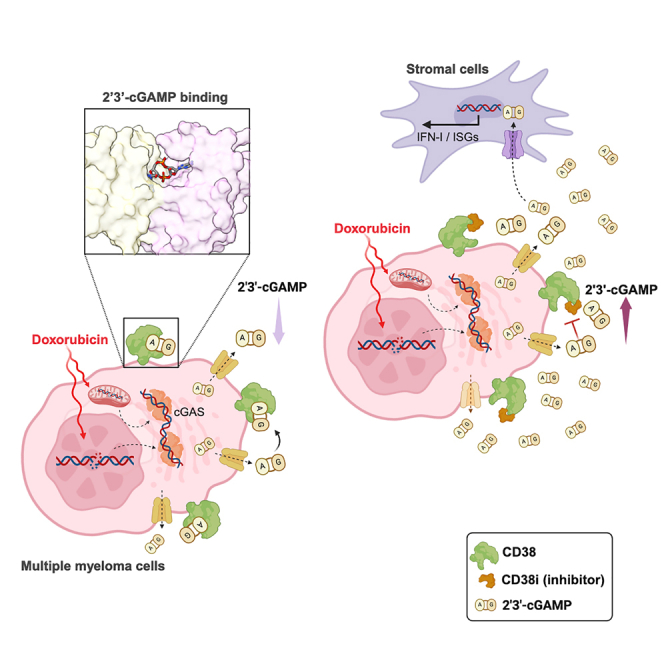

## Introduction

The second messenger 2′3′-cyclic guanosine monophosphate–adenosine monophosphate (2′3′-cGAMP, or simply cGAMP) is a central mediator of the innate immunity, acting at the core of a signaling pathway that sustains and shapes antiviral defense, response to intracellular bacteria, tumor control and rejection, and autoinflammatory manifestations.^1^

cGAMP is synthesized from ATP and GTP by the sensor-enzyme cGAS (cyclic GMP-AMP synthase), which is activated upon binding with double-stranded DNA of viral, bacterial, nuclear, or mitochondrial origin.^2^^,^^3^ cGAMP activates the ER-bound adaptor protein STING (stimulator of interferon genes) either in the cell of origin, or in bystander cells; its movement across the cell membrane is mediated by channel proteins like ABCC1,^4^ LRRC8A/E,^5^ SLC19A1,^6^ SLC46A2,^7^ and gap junctions.^8^ Upon cGAMP binding, STING relocates to the Golgi, oligomerizes and recruits TBK1 (TANK-binding kinase 1), which phosphorylates STING, allowing the binding of IRF3 (interferon regulatory factor 3) and its activation by TBK1. Phospho-IRF3 dimers ultimately translocate into the nucleus, inducing the transcription of type I interferons (IFNs) and several other cytokines and chemokines.^9^ TBK1 can also activate the IKK complex, which phosphorylates the inhibitor IκBα, leading to the activation of NF-κB.^10^

The biology of extracellular cGAMP in the context of cancer represents an encouraging field of investigation, as it was recently implicated in tumor clearance, metastasis control, and radiation-induced antitumor immunity *in vivo*.^11^^,^^12^^,^^13^^,^^14^ In parallel, the research about the regulation of cGAMP stability led to the recognition of ENPP1 (Ectonucleotide pyrophosphatase/phosphodiesterase 1, also known as CD203a or PC-1) as the main cGAMP-degrading ectoenzyme,^15^^,^^16^ whose expression ensures cancerous cells an efficient mechanism of immunoescape.^13^ Possibly because of this function, ENPP1 can foster bone metastatization in breast cancer^17^ and its overexpression has been associated with poor prognosis in high-grade serous ovarian carcinoma.^18^ Moreover, in concert with an array of ectoenzymes acting on chemically related substrates, namely CD38, CD39, and CD73, ENPP1 participates in a series of extracellular reactions that generate the immunosuppressive metabolite adenosine from ATP or NAD.^19^ Adenosine, in virtue of its anti-inflammatory and tolerogenic properties, is currently believed to contribute to myeloma growth and immunoescape in the bone marrow niche.^19^

Interestingly, congenital deficiency of ENPP1 causes a range of syndromes whose principal manifestations are ectopic calcifications and skeletal disorders^20^ but, to date, it has not been convincingly associated with autoinflammation in humans, suggesting the existence of other mechanisms regulating cGAMP immunostimulatory activity. Here, we collected *in cellulo*, *in vitro*, and *in silico* evidence for the role of ADP-ribosyl cyclase/cyclic ADP-ribose hydrolase 1, better known as CD38, in binding and restraining extracellular cGAMP in a model of human multiple myeloma (MM), a malignant neoplasm characterized by a typically high expression of CD38. We show that the genotoxic drug doxorubicin induces MM cell lines to release cGAMP, and that concomitant inhibition of CD38 results in the accumulation of the second messenger in the conditioned medium. In addition, we demonstrate the ability of bone marrow mesenchymal stromal cells (BMSCs) derived from MM patients to respond to exogenous and MM cell-derived cGAMP by upregulating the expression of IFN-β and other genes induced by STING activation. Since extracellular cGAMP has proven to be critical for the control of several malignancies, we speculate that its sequestration by CD38 may represent a general mechanism to attenuate STING signaling in CD38-expressing tumors. We used molecular docking and extensive molecular dynamics (MD) simulations to probe the structure of cGAMP-CD38 complex and then quantitatively analyzed all potential interactions between cGAMP and CD38 to unveil the conformational changes occurring upon ligand binding. *In vitro* experiments finally demonstrate that cGAMP is able to stably interact with its catalytic site, thus corroborating the hypothesis that CD38 may act as a cGAMP decoy receptor.

## Results

### Doxorubicin strongly increases the release of extracellular cGAMP by MM cells

**Figure 1:**
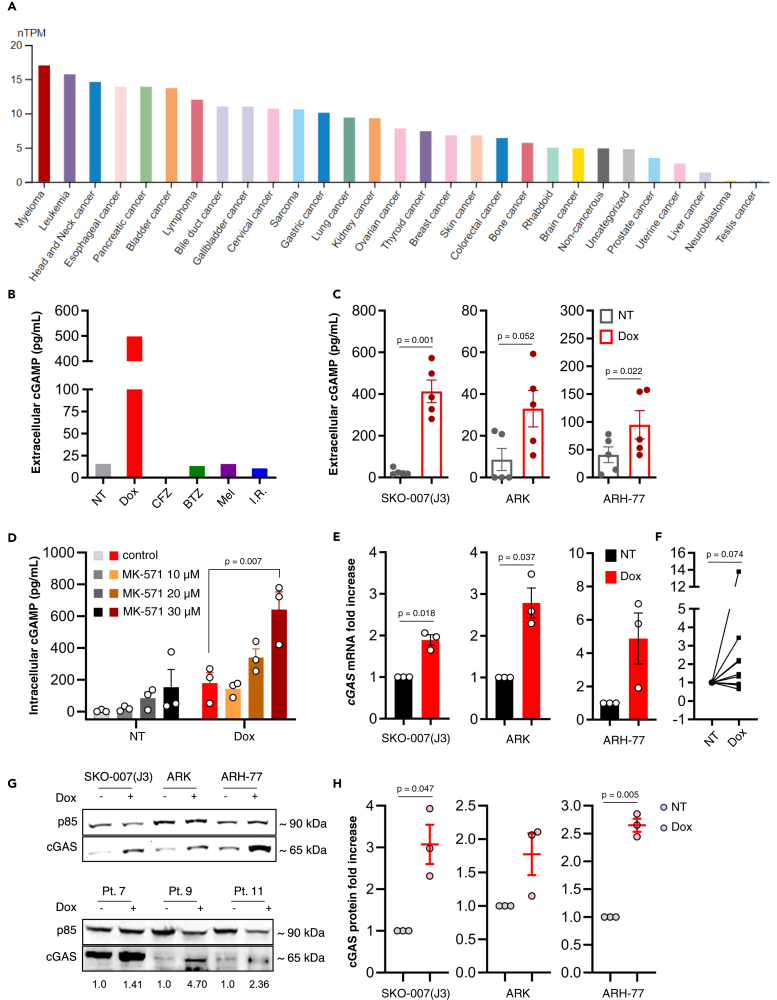
Doxorubicin treatment increases the extracellular release of cGAMP by MM cells (A) Expression of cGAS mRNA (as normalized Transcript Per Million cells) across human cancer cell lines of different origin, grouped by category. Image credit: The Human Protein Atlas (available at: https://www.proteinatlas.org/ENSG00000164430-CGAS/cell+line). (B) The cell line SKO-007(J3) was treated for 24h with Dox 0.2 μM, carfilzomib (CFZ) 18 nM, bortezomib (BTZ) 2 nM, melphalan (MEL) 1 μM and ionizing radiation 25 Gy (I.R.); the medium was then replaced with serum-free medium; after additional 24 h, cGAMP in the CM was quantified through competitive ELISA. (C) SKO-007(J3), ARK and ARH-77 cells were treated for 24 h with Dox 0.2 μM (SKO-007(J3)) or 0.1 μM (ARK and ARH-77), the medium was then replaced with serum-free medium; after additional 24 h, cGAMP in the CM was quantified through ELISA. Statistical significance was calculated using two-tailed paired Student’s T test. (D) SKO-007(J3) cells were treated for 24 h with Dox 0.2 μM; the medium was then replaced with serum-free medium in presence or absence of MK-571 10 μM, 20 μM or 30 μM; after additional 24 h, cGAMP in the cellular lysate (50 μL of lysate for 0.5 · 10^6^ cells) was quantified through ELISA. Statistical significance was calculated using one-way ANOVA with Dunnett’s test for multiple comparisons. (E) SKO-007(J3), ARK, ARH-77 cells and (F) primary MM cells (patients 1 to 10 of Table 1) were treated with Dox for 48 h (for the cell lines concentration is indicated in the aforementioned section; for primary PCs Dox 0.2 μM) expression of *cGAS* gene was measured by real-time PCR and normalized with *GAPDH*. Statistical significance was calculated using one sample T test (for cell lines) and Wilcoxon signed rank test (for primary MM cells). (G) Western blot analysis of cGAS protein in total cellular lysate of untreated and Dox-treated (48 h) SKO-007(J3), ARK, ARH-77 (top panel) and primary MM cells (bottom panel; patients 7, 9, 11, from left to right). The densitometry was calculated by normalizing with PI3K subunit p85. (H) Experimental triplicate of western blot analysis of cGAS protein in MM cell lines. Statistical significance was calculated using one sample T test. Error bars represent SEM.

Human MM cell lines express *cGAS* mRNA at the highest level compared to other types of cancer cell lines^21^ (Figure 1A), suggesting their intrinsic capacity to synthesize large amounts of cGAMP, and making MM, in principle, a suitable tumor model to study its biology. In spite of this, there are currently no indications on the ability of MM cells to export cGAMP; we therefore quantified through ELISA the amount of cGAMP released in serum-free conditioned medium (CM) by the human MM cell line SKO-007(J3) upon different types of stimulation. Treatment of SKO-007(J3) cells with the genotoxic drug doxorubicin (Dox) for 24 h induced a strong release of extracellular cGAMP, whereas treatment with the proteasome inhibitors (PIs) carfilzomib and bortezomib, the alkylating agent melphalan, or ionizing radiation (I.R.), at the doses tested, did not (Figure 1B). This was partly expected, since Dox can reportedly activate cGAS by causing the leakage of mitochondrial DNA in the cytoplasm.^22^ On the contrary, the fact that I.R. did not boost cGAMP export was surprising and in contrast with previous studies focused on cancer cells from solid tumors,^14^^,^^16^ suggesting that the ability of releasing cGAMP is both stimulus- and cell type-dependent. PIs like bortezomib and carfilzomib were shown to induce STING-dependent immunogenic cell death in MM,^23^ but in our model did not enhance extracellular cGAMP release.

ARK and ARH-77 MM cells released cGAMP at much lower levels upon Dox treatment (Figure 1C), whereas for other cell lines tested (multiple myeloma LP-1 and U266, NK leukemia NKL and myeloid leukemia THP-1) no detectable levels of extracellular cGAMP, both basally and upon Dox treatment, were found in the CM.

The ATP-dependent transporter ABCC1, known to mediate the export of drugs, including doxorubicin, has been recently indicated as the major cGAMP exporter.^4^ Accordingly, addition of the ABCC1 inhibitor MK-571 to untreated and Dox-treated SKO-007(J3) resulted in accumulation of intracellular cGAMP in a dose dependent fashion, suggesting that ABCC1 is responsible, to some extent, for cGAMP export in MM cells (Figure 1D). This, however, does not exclude a possible contribution of passive release through channel proteins like VRACs. Moreover, Dox treatment generally increased the expression and protein levels of cGAS in SKO-007(J3), ARK, ARH-77 and, albeit much more variably, in primary malignant plasma cells (PCs) from a fraction of MM patients at disease onset (Table 1), as measured by real-time PCR and western blot analysis (Figures 1E–1H), indicating that Dox can amplify the production and release of cGAMP by triggering a stress response that regulates the amount of cGAS protein at transcriptional level.

**Table 1:** Clinical parameters of MM patients

N°	Sex	Age	Disease stage	% PCs in BM
1	M	59	onset	36%
2	M	55	onset	27%
3	M	72	onset	32%
4	F	70	onset	38%
5	F	64	onset	69%
6	M	90	onset	40%
7	F	82	onset	35%
8	M	61	relapse	45%
9	M	67	onset	50%
10	F	84	onset	19%
11	M	84	relapse	17%
12	F	86	onset	3%
13	F	59	onset	4%
14	M	76	onset	9%
15	M	83	onset	6%
16	F	89	smoldering MM	17%
17	M	75	MGUS^a^	2%
18	F	76	MGUS	15%
19	M	81	onset	24%
20	F	77	relapse	39%
21	F	83	onset	43%
22	M	59	relapse	1%
23	F	66	relapse	65%
24	M	84	onset	30%
25	M	82	relapse	5%
26	M	78	onset	31%
27	M	61	relapse	45%
28	M	58	onset	60%

a Monoclonal Gammopathy of Undetermined Significance.

Of note, and in line with previous reports,^24^^,^^25^ we noticed that high concentration of exogenous cGAMP and, with lower efficacy, the bacterial analog 3′3′-cGAMP, triggered apoptosis in SKO-007(J3), the cell line releasing the highest amount of cGAMP among those tested. Conversely, longer exposure at a lower dose increased senescence-associated β-galactosidase activity, a typical marker of cellular senescence (Figures S1A and S1B). On this basis, it is tempting to speculate that MM cells may export cGAMP as a mechanism to avoid the cytotoxic or cytostatic effects of self-STING overstimulation.

### Bone marrow mesenchymal stromal cells secrete IFN-β in response to exogenous cGAMP

Several works established the existence of complex interactions mediated by soluble cues and cell-to-cell contacts between malignant PCs and BMSCs.^26^^,^^27^ However, the involvement of STING signaling in this crosstalk is yet to be elucidated. Moreover, it is not known whether BMSCs can uptake and respond to extracellular cGAMP.

**Figure 2:**
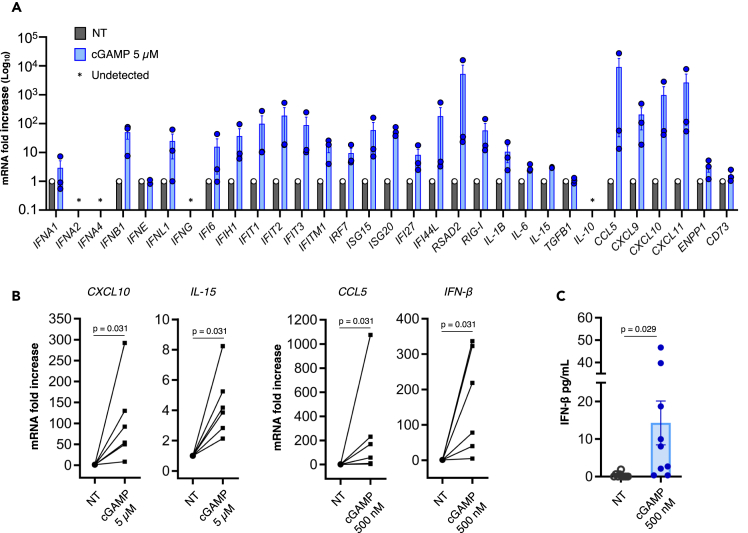
BMSCs from MM patients respond to exogenous cGAMP by implementing a type I IFN-mediated transcriptional response accompanied by IFN-β secretion (A) BMSCs from three MM patients at disease onset (patient n° 28, 29, 30) were treated with cGAMP 5 μM in serum-free medium for 16 h; the expression of the 32 genes indicated in the figure (normalized with *GUSB*) was measured by real-time PCR with a custom TaqMan array plate. (B) BMSCs from MM patients (12–22 of Table 1) were treated with cGAMP 5 μM or 500 nM in serum-free medium for 16 h; the expression of *CXCL10*, *IL-15* (5 μM) and *IFN-β*, *CCL5* (500 nM) was measured by real-time PCR and normalized with *GAPDH*. Statistical significance was calculated using the Wilcoxon signed rank test. (C) IFN-β secretion upon treatment with cGAMP 500 nM was measured by ELISA (patients 1, 7, 12, 13, 14, 15, 16, 17, 18, and 22 of Table 1). Statistical significance was calculated using two-tailed unpaired Student’s T test. Error bars represent SEM.

Therefore, BMSCs from MM patients at different disease stages (Table 1) were isolated by exclusion of non-adherent cells and validated using multiparametric flow cytometry;^27^ cells in serum-free medium were then treated with cGAMP 5 μM for 16 h. The expression of *IFN-β*, several ISGs and chemokines was strongly induced by cGAMP, as measured by real-time PCR (Figure 2A), indicating that BMSCs can uptake soluble cGAMP from the extracellular space, implementing a robust STING-mediated transcriptional response. We confirmed this observation in additional patients on selected genes of particular relevance in the context of antitumor innate immunity (*IFN-β*, *CCL5*, *CXCL10*, and *IL-15*, Figure 2B); of note, a 10-fold lower concentration of cGAMP (500 nM) was sufficient to potently activate the transcription of *IFN-β* and *CCL5*, and to induce the secretion of relevant amounts of IFN-β protein (Figure 2C).

### Inhibition of CD38 results in extracellular cGAMP accumulation

Blocking ENPP1 activity has proven to be an effective strategy to stabilize extracellular cGAMP.^28^ However, flow cytometric measurement of surface ENPP1 on SKO-007(J3), ARK and ARH-77 showed that these cell lines do not express ENPP1 at detectable levels, not even upon Dox treatment (Figure S2A). On the other hand, primary PCs from MM patients express variable levels of ENPP1 on the plasma membrane, ranging from undetectable to low (Figure S2B). Nonetheless, some authors reported relatively high expression of ENPP1 on PCs from MM patients and PC-derived extracellular vesicles.^29^

Since MM cells typically express CD38, an ectoenzyme that acts on substrates structurally and chemically similar to those of ENPP1, we tested whether CD38 inhibition may impact the stability of extracellular cGAMP. Among our cell lines, SKO-007(J3) and ARH-77 express low surface levels of CD38, while ARK cells express very high CD38 surface density (Figure S2C).

**Figure 3:**
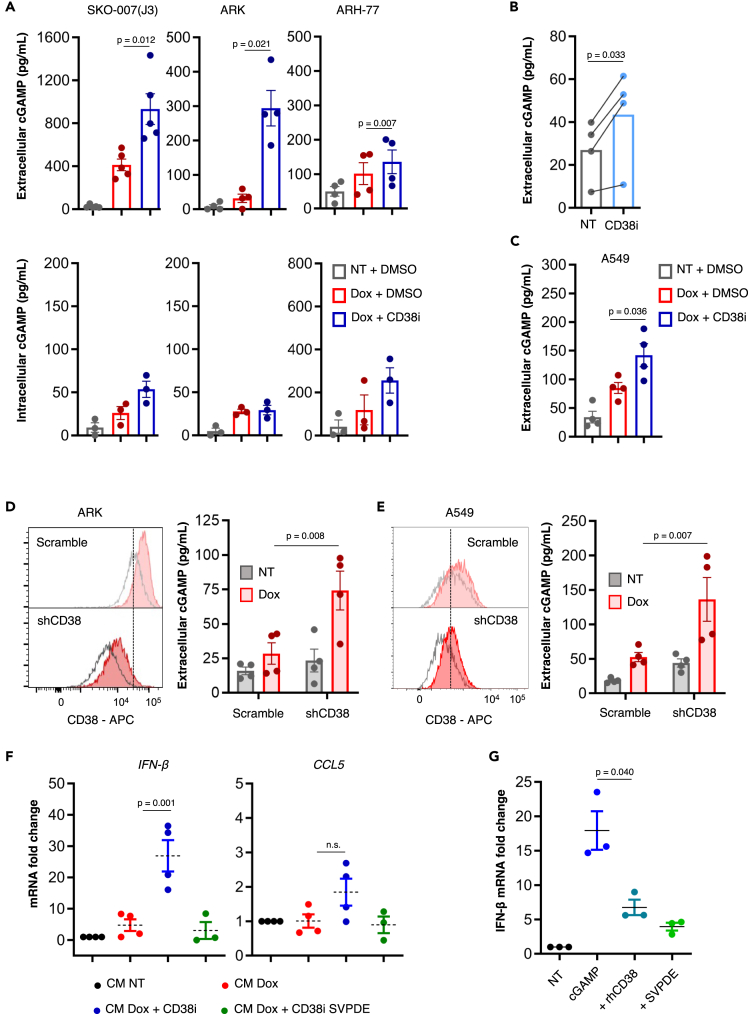
Inhibition of CD38 on Dox-treated MM cells causes accumulation of extracellular cGAMP (A) Cells were treated with Dox for 24 h as already described; the medium was then replaced with serum-free medium containing DMSO or CD38i (10 μM for SKO-007(J3) cells, 20 μM for ARK and ARH-77 cells); after additional 24h, cGAMP in the CM (top panel) and in the cellular lysate (50 μL of lysate for 0.5 · 10^6^ cells, bottom panel) was quantified through competitive ELISA. Statistical significance was calculated using one-way ANOVA with Tukey’s test for multiple comparisons. (B) Primary PCs from four MM patients at disease onset (1–4 of Table 1) were treated for 48 h with CD38i 10 μM or DMSO in serum-free medium enriched with 20 μM MnCl_2,_ 20 ng/mL IL-3 and 2 ng/mL IL-6; the concentration of cGAMP in the CM was then quantified through ELISA. Statistical significance was calculated using two-tailed paired Student’s T test. (C) ARK cells were transduced with a lentiviral vector carrying short hairpin RNA targeting CD38 (shCD38) or non-targeting (scramble). After selection in G418, surface expression of CD38 was measured by flow cytometry. (D and E) ARK and A549 shCD38 and scramble control were stimulated with Dox and subsequently cGAMP was measured in CM as described previously. Statistical significance was calculated using two-way ANOVA with Bonferroni correction for multiple comparisons. (F) BMSCs from MM patients (12–15 of Table 1) were incubated for 16 h with the CM from SKO-007(J3); the transcription of *IFN-β* and *CCL5* was then quantified through real-time PCR (normalized with *GUSB*). Statistical significance was calculated using one-way ANOVA with Tukey’s test for multiple comparisons. (G) THP-1 cells were incubated for 6 h with serum-free medium containing cGAMP 100 nM, pre-incubated with rhCD38 10 nM or nuclease-free water (vehicle) for 1 h at 37°C. The induction of *IFN-β* was then measured by real-time PCR. Statistical significance was calculated using one-way ANOVA with Tukey’s test for multiple comparisons. Error bars represent SEM.

Strikingly, when a cell-permeable inhibitor of CD38 (CD38i, 78c) was used in combination with Dox, a higher concentration of cGAMP was detected both in the CM and, less distinctly, in the intracellular compartment of SKO-007(J3), ARK and ARH-77 cells compared to Dox treatment alone (Figure 3A). In addition, Dox generally increased CD38 protein and mRNA expression in MM cell lines, whereas it had negligible effect on primary PCs (Figures S2C and S2D).

Primary PCs cultivated *ex vivo* released lower levels of cGAMP compared to MM cell lines, while Dox treatment had unpredictable effect on their viability; for this reason, in order to detect a possible difference in cGAMP extracellular level upon CD38 inhibition, we treated PCs with DMSO or CD38i in the absence of Dox, in serum-free medium enriched with IL-3, IL-6, and MnCl_2_, which boosts cGAS activation.^30^ Such conditions allowed us to detect a higher concentration of extracellular cGAMP in the presence of CD38i from PCs of four patients at disease onset (Figure 3B).

These findings were confirmed in CD38-expressing lung adenocarcinoma cell line A549 (Figure 3C), demonstrating that the inverse correlation between extracellular cGAMP levels and CD38 activity is not limited to myeloma cells.

To rule out possible off-target effects of the inhibitor, we transduced both ARK (the MM cell line expressing the highest surface density of CD38) and A549 cells with a lentiviral construct carrying a short hairpin RNA targeting CD38 (shCD38) or non-targeting (scramble). After selection in G418 and validation by flow cytometry, we treated the cells with Dox and measured cGAMP concentration in the CM as described previously. The higher levels of cGAMP detected in the CM of Dox-treated shCD38 cells compared to the scramble control (Figures 3D and 3E) were in line with the results obtained with CD38i, confirming the specific effect of the inhibitor.

Next, to verify whether the amount of extracellular cGAMP released by Dox-treated cells incubated with CD38i was sufficient to exert a biological response, BMSCs from MM patients were incubated for 16 h with CM from SKO-007(J3) cells. Subsequently, transcriptional induction of *IFN-β* and other genes induced by the STING pathway was measured by real-time PCR. At concentrations of cGAMP reached in the CM, however, only two genes among those tested were detectably induced, namely *IFN-β* and *CCL5.* As negative control, CM was pre-treated with snake venom phosphodiesterase (SVPDE), which efficiently hydrolyzes cGAMP,^31^ for 40 min at 37°C to fully inactivate soluble cGAMP. BMSCs treated with CM from CD38-inhibited SKO-007(J3) expressed higher mRNA levels of *IFN-β* and, to a lesser extent, of *CCL5* and compared to BMSCs incubated with CM of SKO-007(J3) treated with Dox alone (Figure 3F). Further, pre-treatment of the CM with SVPDE abrogated this effect (Figure 3F), demonstrating that cGAMP is responsible for the transcriptional induction.

The accumulation of extracellular cGAMP upon CD38 inhibition or knockdown suggested to us that the ectoenzyme could be directly implicated in its stability or availability. We hypothesized that CD38 could directly dampen cGAMP activity either through enzymatic degradation or by sequestration following direct binding. To test this hypothesis and to exclude any indirect effect of CD38 function on cGAMP production, we incubated THP-1 cells, used as a reporter cell line for testing the presence of soluble cGAMP,^14^ for 6 h in serum-free culture medium enriched with cGAMP, pre-incubated with human recombinant CD38 protein (rhCD38) or SVPDE; the expression of *IFN-β* by THP-1 cells was then quantified. As shown in Figure 3G, pre-incubation with rhCD38 significantly reduced the ability of cGAMP to induce the expression of *IFN-β* in THP-1 cells, an observation compatible with both degradation and sequestration.

Previous reports point at CD38 as an interferon-inducible gene (ISG).^32^^,^^33^ Accordingly, cGAMP treatment of NK cell-derived leukemia cell line NKL and monocytic leukemia cell line THP-1 resulted in a dose-dependent increase of CD38 surface expression (Figures S2E and S2F), whereas a similar effect was not observed on ARK MM cells, suggesting that the response could be cell type-dependent. Admitting the negative regulation of cGAMP by CD38, these observations support the hypothesis of a cGAMP-type I IFN-CD38 negative feedback loop.

Collectively, these results show that CD38 can directly restrain the biological activity of cGAMP.

### Atomistic molecular modeling of CD38-cGAMP complex

**Figure 4:**
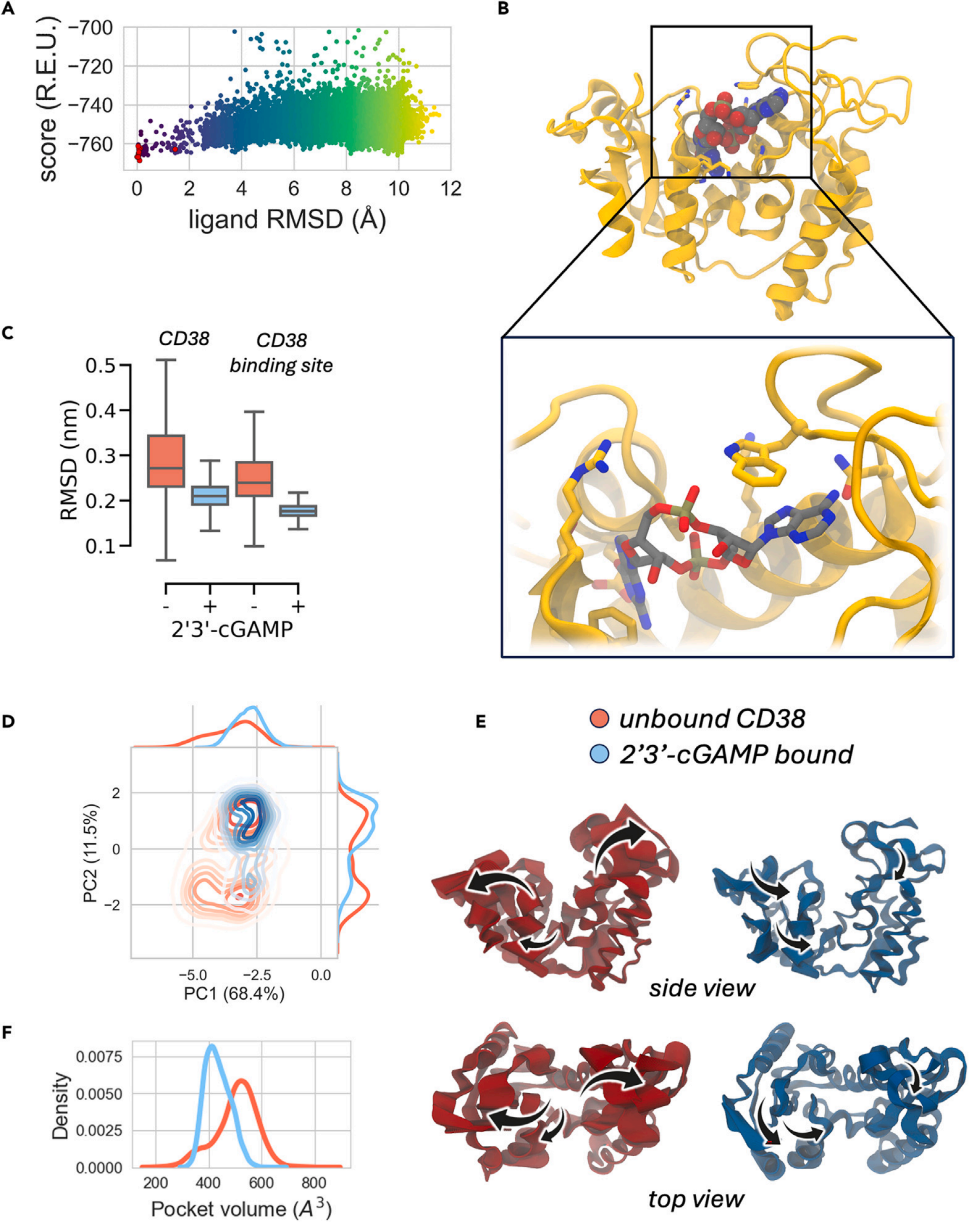
Molecular docking and multi-copy μs-long MD simulations reveal stable interactions of cGAMP with CD38 and demonstrate that cGAMP binding leads to significant CD38 global rigidification (A) CD38-cGAMP complex total energy score was plotted against cGAMP unsuperimposed root-mean-square deviation (ligand RMSD), from which the top 10 binding models (red dots) were selected. The model with lowest binding energy was set as the reference. (B) Predicted binding mode of cGAMP to CD38. (C) Trajectory median backbone RMSD of unbound (red) and bound (blue) CD38. The increased conformational rigidity of the CD38-cGAMP complex compared to the unbound CD38 is demonstrated by a median backbone RMSD of ∼3 Å and ∼2 Å along the simulation time, respectively (left boxplot). The overall rigidification of the protein in the bound state is reflected in the active site as well (right boxplot). Error bars denote SD. (D) Principal Component Analysis (PCA) of unbound (red) and bound (blue) CD38. The projection to the first two PCA-eigenvectors is based on the Cα of all simulated ensembles. Distributions for the projection on the first and second eigenvectors are plotted previously and on the right of the corresponding plots. (E) Collective motions corresponding to the first PC of unbound and bound CD38. Comparison of the motion described by eigenvector 1 from the unbound (red) and bound (blue) MD ensembles. Motions are illustrated as linear interpolations between the extreme projections of the structures onto the eigenvector 1 are indicated by black arrows. The direction of the arrow in each Cα atom represents the direction of motion, while the length of the arrow characterizes the movement strength. A schematic of the described motions is presented (see also Videos S1 and S2). (F) Catalytic pocket volume distributions for unbound (red) and bound (blue) simulations.

In order to investigate the hypothesis of cGAMP as a putative ligand of CD38, *in silico* molecular docking and MD simulations were employed here to explore the binding orientation of cGAMP within the active site of the extracellular domain of CD38 (residues 43–300).^34^ Site-specific docking, employing an all-atom fully flexible representation of both protein and ligand, allowed us to identify both the protein residues critical for the interaction and the spatial orientation of the ligand. The final docking pose (Figures 4A, 4B, and S3A) was chosen as described in detail in the molecular docking section of computational methods.

To refine the cGAMP-CD38 complex model in explicit water, to assess its stability,^35^^,^^36^ and to gain structural and mechanistic insights into ligand—receptor interaction, extensive multi-copy MD simulations in the microsecond timescale were performed for CD38-cGAMP complex as well as for CD38 unbound protein. CD38 backbone root-mean-square deviation (RMSD) and root-mean-square fluctuation (RMSF) data, averaged along the MD trajectories, suggest that the presence of the cGAMP reduces the conformational space explored by CD38 (Figures 4C, S3B, and S3C). This was confirmed by means of RMSD cluster analysis (Figures S3D and S4A). Only minimal differences in terms of gyration radius were detected between unbound and bound states, suggesting that cGAMP slightly affect the global compactness of CD38 (Figure S4B). Finally, the low binding site RMSD (0.87 Å) between unbound and bound representative structures suggested a model of conformational selection binding between cGAMP and CD38.

To better understand how cGAMP binding might impact CD38 dynamics and in order to filter out functionally relevant collective motions from local “noise”, we performed principal-component analysis (PCA) on our MD simulations. This method allows identifying the key components (i.e., atoms) of a system that are responsible for large-scale motion endowed with a relatively long timescale. In our case, using such an approach, we detected the most relevant slow motion of the systems along the MD simulations and provided a graphical representation of the main conformational rearrangements of CD38 both in the bound and unbound states^37^(Figures 4D and 4E).

Changes in the intensity of motions captured by the first eigenvector are depicted in the porcupine plots of Figure 4E: the unbound state of CD38 exhibited a pronounced *inter-domain clamping* motion, while cGAMP binding produced a significant decrease in the *inter-domain clamping* motion (see also Video S1, PC1 of the unbound CD38, related to Figure 4; and Video S2, PC1 of the cGAMP—bound CD38, related to Figure 4). The second eigenvector described a *twisting* motion that principally involves the middle loop (G245-S250) in the C-terminal domain (Figure S4C). The *twisting* motion was altered upon cGAMP binding: in the unbound state eigenvector 2 described the opening of the C-terminal domain, while in the bound state the middle loop (G245-S250) and the α4 helix sampled reciprocally opposite concerted motion, in terms of directionality, to better accommodate cGAMP in the active site. As a result, the motion strength of CD38 in the bound state is highly weakened upon cGAMP binding.

Video S1. PC1 of the unbound CD38, related to Figure 4

Video S2. PC1 of the cGAMP – bound CD38, related to Figure 4

To evaluate the impact of cGAMP on the active site conformational plasticity in greater detail, we monitored the changes on the active site volume along the MD simulations (Figures 4F and S4D). CD38 fluctuates between two states in the unbound form as shown in Figure S4D and by the distribution of the binding pocket volume shown in Figure 4F, displaying wide fluctuations from 188 to 859 A^3^ and an average volume of 505 ± 80 A^3^. Moreover, the D175-C180, G210-I215, and middle loop (G245-S250) loops overhanging the binding cavity exhibits high flexibility (Figures 4E and S3C) in the unbound form. This is in accordance with the DynaMine score higher than 0.7 (indicating highly dynamic flexible protein backbone regions) for these loops, as reported in the PDBe-KB (PDBe-KB: https://www.ebi.ac.uk/pdbe/pdbe-kb/proteins/P28907/structures).^38^

The active site cavity volume is stabilized at 435 ± 47 A^3^ (ranging from a minimum value of 315 to a maximum of 672 A^3^) by cGAMP binding, leading to a narrow cavity conformation, as shown in Figure 4F. Upon binding of cGAMP, the flexibility of the cavity overhanging loops is reduced and CD38 sampled a lower number of cavity conformations with smaller volumes than the unbound form (Figures 4F and S4D).

Taken together, this evidence suggests that CD38 positions cGAMP like a wedge between the two domains (Figures 4B and S4A) without signs of unbinding or significant reorientations (Figure S4E) —likely following a conformational selection model of interaction—stabilizing the more rigid conformation of the protein, especially regarding the catalytic pocket.

### Molecular interactions between cGAMP and CD38 active site

During microseconds MD simulations, the adenine and guanine moieties of cGAMP are accommodated within the N-domain portion of the binding pocket (here defined as N-pocket) and the C-domain portion of the pocket (here defined as C-pocket), respectively (Figure 4B).

**Figure 5:**
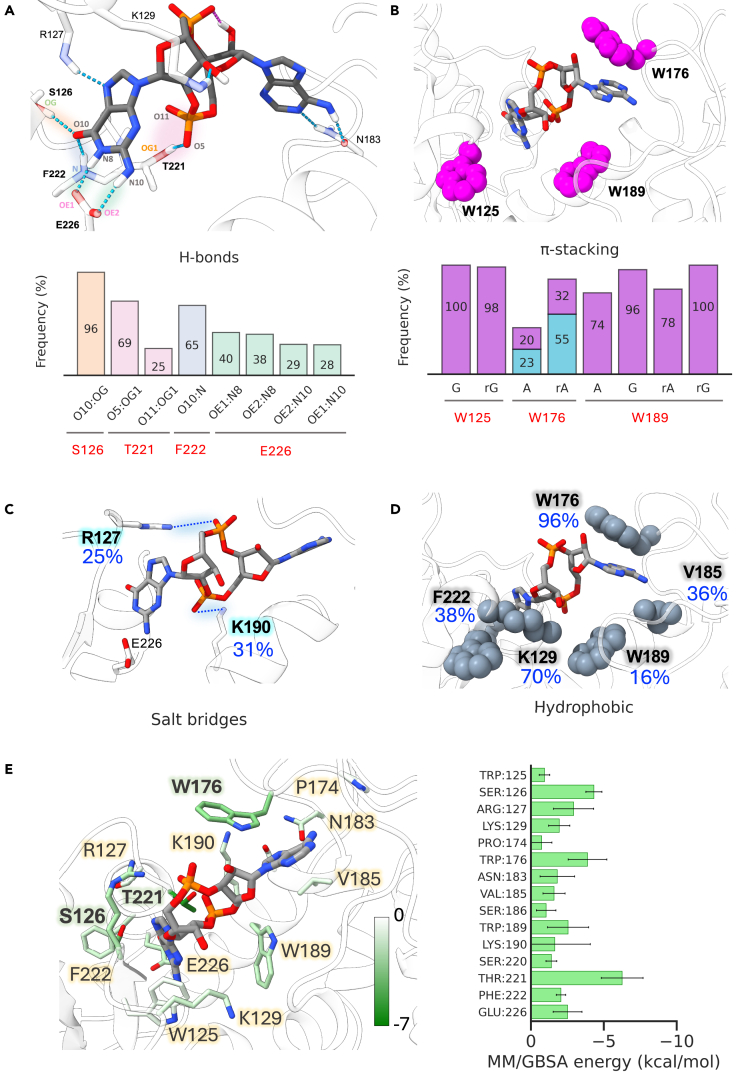
Molecular interactions between CD38 catalytic pocket and cGAMP (A–D) Statistical distribution (% of the total simulation time) of the interactions between CD38 residues and cGAMP ligand. For the hydrogen bonds and salt bridges analyses, given the high number of interactions observed, only the interactions with a persistence higher than 25% of the simulation time have been reported to limit the description to the most relevant ones. For the π—stacking interactions, the contacts observed for more than 20% of the simulation time are reported. Values are reported by averaging the results from the three replicate simulations for both unbound and bound systems. (E) Per-residue Molecular mechanics generalized Born and surface area (MM/GBSA) binding affinity estimate (right) and corresponding hot spot residues (left) are illustrated. The binding free energy from different residues located within a 5 Å radius from the ligand is shown. Only residues contributing with more than *k*T (∼0.6 kcal/mol at 300 K) are reported. Values are reported by averaging the results from the three replicate simulations for both unbound and bound systems.

The adenosine moiety of cGAMP is accommodated in the N-pocket defined by K190, W176, N183, V185, T158, S186, and W189, in a similar manner to that of pA(3′,5′)pG (pApG) of ENPP1 protein in complex with 3′3′-cGAMP.^39^ The adenine base is sandwiched between W176 and W189, and forms hydrogen bonds with N183, S186, and T158 in a base-specific manner (Figures 5A and 5B). The guanosine moiety of cGAMP is accommodated in the C-pocket, defined by R127, S126, F222, K129, W125, and E226. The OH group of the guanosine (O10) forms a very stable hydrogen bond with S126 (96% of the total frames) (Figure 5A), while the N and the NH group of the guanosine (N8 and N10) are involved in hydrogen bonds with the catalytic residue E226 (Figure 5A). The guanine base is stacked with W125 (Figure 5B). In addition to E226, cGAMP interacts with the substrate binding motif (W125, S126, R127, T221, and F222) of CD38 identified previously^40^ (Figure 5A). A persistent cGAMP intra-molecular hydrogen bond between the phosphate (O13 oxygen) and the hydroxyl moieties (H15 hydrogen) in the adenine ribose were detected in 92% of the analyzed frames (Figure 5A).

As already reported in the ENPP1—2′3′-cGAMP complex model,^39^ the guanine-linked ribose of the 2′3′-cGAMP bound to CD38 adopts the C2′-endo conformation for 78.4% of the MD simulation (Figure S4F) in which its 3′-oxygen atom forms hydrogen bonds with K129, rather than N259 in ENPP1. The relative binding free energy (ΔG_binding_) underpinning the recognition of cGAMP and CD38 was estimated by means of the molecular mechanics generalized Born model and surface area (MM/GBSA) method (see computational methods for details). The details of the different energy components of the binding affinity between CD38 and cGAMP are summarized in Table 2.

**Table 2:** Energetic components of the relative binding free energy for cGAMP-CD38 complex

Energy component	Mean	SD
ΔE_ele_	−224.8	39.8
ΔE_vdw_	−49.0	8.0
ΔG_pol_	227.4	35.7
ΔG_np_	−5.6	0.7
ΔG_gas_	−273.9	41.5
ΔG_sol_	221.8	35.5
ΔG_bind_	−52.1	10.4

The high affinity of cGAMP for CD38 (calculated relative ΔG_binding_ = – 52.1 ± 10.4 kcal/mol) was supported by the detailed atomic analysis of polar, non-polar, and electrostatic interactions discussed previously. This is consistent with the nature of key residues stabilizing the cGAMP molecule in the pocket, which are almost exclusively polar (T221, S126, and R127) or aromatic (W176, W189, and W125). A large fraction of the hydrophobic binding energy of cGAMP may be due to a face-to-face π−π stacking between the ribose of adenosine and the indole of W176 (Figure 5E) as detected by time-course analysis of the total relative ΔG_binding_ (Figures S4G and S4H). These data laid the foundations for further *in vitro* experiments detailed in the following text.

### *In vitro* experiments corroborate *in silico* predictions

The simulation results were corroborated by direct binding experiments performed using fluorescence intensity quenching (FQ) of CD38 tryptophan residues and by microscale thermophoresis (MST). Containing eight tryptophan residues, CD38 has been previously analyzed through FQ for studying enzyme-ligand interactions and for determining the dissociation constants.^41^^,^^42^

**Figure 6:**
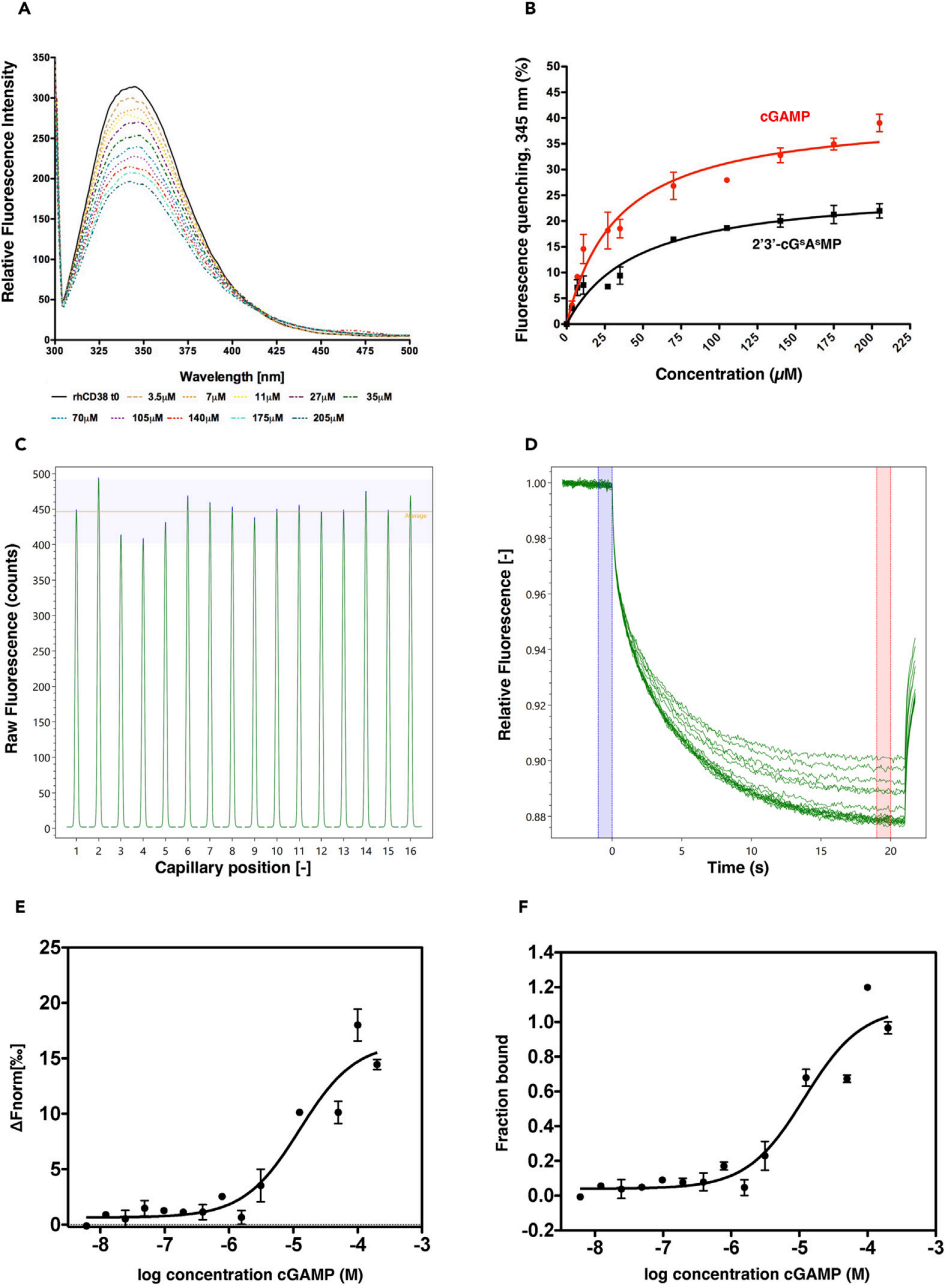
Tryptophan intrinsic fluorescence measurements and MST analysis show the interaction between CD38 and cGAMP (A) Fluorescence quenching titration of rhCD38 (1 μM) with increasing cGAMP (0–205 μM) in 50 mM Phosphate buffer pH 7.4 and 50mM NaCl at 25°C upon excitation at 295 nm. The black curves represent the emission spectrum of rhCD38 in the absence of ligands. The colored curves show the progressive decrease in fluorescence intensity of rhCD38 at the emission maximum (345 nm) upon the addition of cGAMP. (B) Plot of normalized fluorescence signal at 345 nm expressed as percentage of quenching versus the concentrations of dinucleotides. By applying a nonlinear regression fitting, dissociation constants values (K_D_) of 33.2 ± 5.4 μM and 46.3 ± 9.7 μM were calculated for cGAMP (red) and 2′3′-cG^s^A^s^MP (black), respectively. (C) Capillary scan of MST analysis. (D and E) MST traces and dose–response curves reported as ΔF_norm_ = normalized fluorescence. Error bars indicate the standard deviation of two independent replicates (*N* = 2). (F) MST dose-response curves reported as Fraction Bound. A dissociation constant (K_D_ ± SD) of 8.1 ± 2.5 μM and signal-to-noise ratios (S/N) of 8.7185 were estimated using the software MO Affinity Analysis, v 2.27.

FQ measurements were performed exposing the ectodomain of recombinant human CD38 (rhCD38) to increasing concentrations of cGAMP. The fluorescence emission spectrum of rhCD38 revealed a peak at around 345 nm whose intensity was dose-dependently decreased in presence of cGAMP (Figure 6A). Control experiments with cGAMP alone showed that the intrinsic fluorescence emission of the dinucleotide was negligible and had no substantial impact on the experiments (Figure S5A). The ENPP1-resistant analogue 2′3′-cG^s^A^s^MP, reported by Li and colleagues,^15^ also induced fluorescence quenching of rhCD38, albeit to a slightly lesser extent (Figure S5B). The value of the dissociation constant (K_D_) for the interaction rhCD38-cGAMP was estimated by applying a non-linear regression analysis on the fluorescence decrease as a function of concentration assuming the presence of a single binding site. The estimated K_D_ was 33.2 ± 5.4 μM, while for the resistant analogue was 46.3 ± 9.8 μM, indicating a somewhat similar affinity for the enzyme compared to the natural cGAMP (Figure 6B). The validity of the approach was assessed using β-NAD, the best-known substrate of CD38.^41^ β-NAD also induced fluorescence quenching and exhibited a K_D_ of 28 ± 9.4 μM (Figures S5C and S5D), a value comparable with that reported in literature^42^^,^^43^ and, importantly, of the same order of magnitude as cGAMP.

These observations indicate that cGAMP and the structurally similar analog 2′3′-cG^s^A^s^MP can physically interact with rhCD38, accessing a protein cavity where they interact with tryptophan residues.

The binding of cGAMP to rhCD38 was further confirmed using MST, by which we estimated a K_D_ of 8.1 ± 2.5 μM (Figures 6C–6F), that is in the same range of that determined by FQ. Using MST, we similarly determined the binding affinity of cGAMP for ENPP1 (Figures S6A–S6D), estimating a K_D_ of 1.1 ± 0.4 μM.

We next tested whether rhCD38 was able to hydrolyze cGAMP *in vitro* exposing the dinucleotide (200 nM, 37°C) to the enzyme at a 50:1 M ratio. The disappearance of cGAMP and formation of the main products expected from hydrolyzation, which are linear GAMP, AMP, GMP, adenosine, guanosine, adenine, and guanine, were tested by high resolution LC-MS analyses integrating the peaks obtained extracting the corresponding mass peaks (Table S1). Analysis of the MS data showed that cGAMP was not transformed under these conditions and that none of the expected products was revealed after up to 180 min (Figures S7A–S7C; Table S1), suggesting that cGAMP is sequestered by the protein without the occurrence of an enzymatic transformation. As control, the ability of rhENPP1 to hydrolize cGAMP was assessed by exposing the dinucleotide (200 nM) at 4 nM rhENPP1 and observing the expected formation of AMP and GMP already after 60 min (Figure S8).

Collectively, these observations suggest that cGAMP can directly interact with rhCD38 occupying its catalytic pocket, but it is not transformed into one or more chemical species expected from phospho-ester bond cleavage or guanine and adenine removal.

To further investigate whether cGAMP binds to rhCD38 within or in proximity of its active site, we tested the ability of the dinucleotide to block the hydrolase activity of the enzyme.

We first verified that rhCD38 was able to hydrolyze its substrate ε-NAD in a dose-dependent manner, showing a K_m_ of 56.2 ± 8.9 x 10^−6^ M (Figure S9A). Experiments were then repeated in the presence of increasing concentrations of cGAMP, observing that the dinucleotide dose-dependently inhibited the hydrolase activity up to about 35% (Figure S9B). The CD38 inhibitor 78c, used as positive control, also strongly inhibited the enzyme hydrolase activity (50% at the concentration of 200 nM, data not shown). The data thus show that cGAMP, which apparently is not hydrolyzed by rhCD38, binds the enzyme active site that catalyses the hydrolysis reactions, confirming *in silico* atomistic molecular modeling predictions.

## Discussion

In the present work, we observed for the first time the extracellular release of cGAMP by human myeloma cells, strongly enhanced by the treatment with the genotoxic drug doxorubicin, and we further demonstrated a role of the NAD-glycohydrolase CD38 in the regulation of extracellular cGAMP activity, pointing at its inhibition as a potential strategy to increase extracellular cGAMP concentration for therapeutic purposes.

The novel concept of cGAMP as an extracellular “immunotransmitter”^14^ with powerful immunostimulatory activity demands intensive investigation about its modes of regulation. The main regulatory mechanism identified so far is the enzymatic degradation by ENPP1, which, interestingly, seems to be confined to the extracellular space.^14^^,^^16^ However, considering the potential catastrophic effects of unrestrained cGAMP signaling, in our opinion such mechanism is unlikely to be the sole.

Catabolic ectoenzymes, including ENPP1 and CD38, are often characterized by a remarkable promiscuity toward different (albeit similar) substrates. CD38 and ENPP1 share important similarities; in particular, ADP-ribose (ADPR) is a product of NAD hydrolysis by CD38 and substrate of ENPP1, which hydrolyzes it into AMP.^19^^,^^44^ Moreover, a NAD-degrading activity was originally described for mouse ENPP1 itself.^45^ At the same time, ENPP1 and CD38 present important differences. For example, ENPP1 enzymatic hydrolysis toward ATP and cGAMP shows an optimum at alkaline pH,^15^ whereas the hydrolase and cyclase activities of CD38 toward its known substrates generally have an optimum at neutral or acidic pH,^46^ theoretically closer to that of the TME.

Moving from these considerations, we collected evidence suggesting that CD38 can bind cGAMP into its catalytic pocket and that its inhibition can “free” a significant amount of cGAMP in the extracellular space, thus increasing its biological activity. In particular, we first demonstrated in cellular models that CD38 inhibition or knockdown causes cGAMP to accumulate in the extracellular space; then, aided by detailed molecular docking and MD simulations, we hypothesized a direct binding between the enzyme and cGAMP involving tryptophan residues, hypothesis confirmed by intrinsic fluorescence measurements.

We thoroughly tackled the hypothesis of the enzymatic degradation of cGAMP by CD38; however, our mass spectrometry experiments showed that, at least in our *in vitro* experimental conditions, recombinant CD38 cannot transform cGAMP, in contrast to ENPP1, which can efficiently convert it into AMP and GMP. However, we do not exclude that some particular conditions occurring only *in cellulo* (or, at least, that we were not able to reproduce *in vitro*) can lead CD38 to transform cGAMP into other chemical species. This hypothesis would be more compatible with our estimated K_D_, which is in the typical order of magnitude of substrate-enzyme interactions. In this regard, it is worth noting that CD38 exists in two opposite configurations on the cell membrane, facing either the extracellular or the intracellular environment,^47^ the latter being characterized by micromolar cGAMP concentrations.^16^ Therefore, it is possible that part of the decoy activity of CD38 toward cGAMP actually occurs in the intracellular space. This would be compatible with our observations *in cellulo*, which have been generated with both the cell-permeable CD38 inhibitor 78c and shRNA-mediated knockdown.

Our molecular docking and MD simulations demonstrate that the binding of cGAMP in the catalytic pocket of CD38 is characterized by considerable stability without signs of unbinding or significant reorientations observed along the microseconds MD simulations. Interestingly, 2D projections of unbound and bound states in the essential subspace defined by PCA analysis showed considerable overlap in the most populated conformational substrate, which could indeed represent the CD38 conformation that initially binds cGAMP. Particularly, cGAMP could select a CD38 conformation already sampled from the simulated unbound ensemble, pointing to a conformational selection model of binding.

cGAMP is able to restrict the conformational plasticity of the active site, indicating that it is tightly bound, and it is determining a population shift toward narrow distributions of active site volumes. Moreover, the analysis of the pocket volume distributions showed that the unbound pocket volume density partially encompassed the one computed for the bound state, suggesting a preorganization of the pocket in the unbound state. All available experimental structures of CD38 in the bound state deposited in the Protein DataBank were analyzed to gain insight into potential interactions between the ligand and protein. Our predicted interaction pattern of the bound cGAMP with the active site is in accordance with those of the experimentally determined bound substrates of human CD38 (see results).^48^ It is worth to be noted that the absence of cyclic di-nucleotide ligands co-crystallized with CD38 make the comparison with the experimentally determined bound ligands non-trivial.

CD38-catalyzed hydrolysis determines the cleavage of the N-glycosyl bond in the substrates embedding it deep toward the bottom of the active site pocket, where catalysis occurs.^49^ In this case, ribose establishes direct polar interactions with the E226 carbonyl group. In our CD38-cGAMP model, the catalytic E226 strongly interacts with the guanine base instead of the corresponding ribose, which in turn is stabilized by H-bonds and hydrophobic interactions with K129 and W125 (Figures 5A–5E). Given the intrinsic timescale limit of the MD simulations, it’s difficult to determine if a conformational change in CD38, needed to orient cGAMP in a hydrolysis competent manner, could occur.

The biological relevance *in vivo* of cGAMP sequestration by CD38 is to be demonstrated; however, it is plausible that within the MM niche, where malignant PCs are densely packed and show extremely high surface density of CD38, the restraint of cGAMP activity by CD38 may represent a relevant immunoevasive mechanism. CD38 is also abundantly expressed by most immune cells upon activation;^50^ therefore, the CD38-cGAMP interaction may play a regulatory role in immune cell-highly infiltrated tumor microenvironments, where cGAMP can be released by cancer cells spontaneously or upon treatment.^11^^,^^13^^,^^14^ Further work will be required to unveil the physiological consequences of such interaction in different contexts.

### Limitations of the study

This study was based on human cell lines and BMMCs derived from MM patients and may not be applicable to mouse models, despite the similarity between human and mouse CD38. We acknowledge that it would be of great interest to better define the biological relevance of our finding. However, it would be extremely challenging, with our current knowledge, to evaluate the exclusive effect of the interaction between the multifunctional enzyme CD38 with cGAMP, since deleting CD38 completely, even in an STING-KO background model, may generate confounding effects due to its capacity to also degrade NAD, produce calcium-mobilizing second messengers or act as a counter-receptor for CD31.

The estimated K_D_ for the rhCD38-cGAMP interaction, as we report, is in the micromolar range, which, in principle, is more compatible with that of an enzymatic reaction rather than a decoy activity. Unfortunately, the concentrations at which extracellular cGAMP actually operates in the tumor microenvironment are currently unknown, as unknown is the concentration reached by cGAMP in close proximity to the membrane of the cell that releases it. Techniques such as the ELISA used in the present work can only provide an average estimate of the concentration of analyte accumulated in the highly diluted CM, a condition clearly distant from what happens *in vivo*, where cell density is very high while the space between adjacent cells is minimal. Furthermore, we cannot exclude that the conditions we adopted in our biochemical assays might not reproduce faithfully what occurs *in cellulo* (e.g., specific post-translational modifications of CD38, clustering of the ectoenzyme, presence of cofactors, etc.).

## STAR★Methods

### Key resources table

**Table:** 

REAGENT or RESOURCE	SOURCE	IDENTIFIER
**Antibodies**		

Anti-CD38/APC (clone HIT2)	BD Biosciences	Cat# 560980 RRID: AB_398599
Anti-CD138/FITC (clone MI15)	BD Biosciences	Cat# 347191 RRID: AB_400257
Polyclonal sheep Anti-ENPP1/APC	R&D	Cat# FAB6136A
Polyclonal sheep IgG/APC	R&D	Cat# IC016A
Anti-CD45/APCH7 (clone 2D1)	BD Biosciences	Cat# 560274 RRID: AB_1645479
Anti-CD105/APC (clone 266)	BD Biosciences	Cat# 562408 RRID: AB_11154045
Anti-CD90/PeCy5 (clone 5E10)	BD Biosciences	Cat# 555597 RRID: AB_395971
Anti-CD146/BV395 (clone P1H12)	BD Biosciences	Cat# 752989
Anti-CD106/PE (clone 51-10C9)	BD Biosciences	Cat# 555647 RRID: AB_396003
Anti-CD73/V450 (clone AD2)	BD Biosciences	Cat# 561255 RRID: AB_11151899
Mouse IgG1, κ Isotype Control/APC	BD Biosciences	Cat# 555751
Rabbit anti-human PI3 Kinase p85, N-SH2 domain	Merck Millipore	Cat# ABS233
Rabbit anti-human cGAS (clone D1D3G)	CellSignaling Technology	Cat# 15102

**Bacterial and virus strains**		

DH5α Competent Cells (E. Coli)	ThermoFisher	Cat# 18265017

**Biological samples**		

Human bone marrow mesenchymal stromal cells	Isolated in house	N/A
Human primary multiple myeloma cells	Isolated in house	N/A

**Chemicals, peptides, and recombinant proteins**		

Doxorubicin	Sigma-Aldrich	Cat# D1515
Melphalan	Sigma-Aldrich	Cat# M2011
Bortezomib	Selleckchem	Cat# S1013
Carfilzomib	Selleckchem	Cat# S2853
2’3’-cG^s^A^s^MP	Invivogen	Cat# tlrl-nacga2srs
2’3’-cGAMP	Invivogen	Cat# tlrl-nacga23-02
MK-571	Sigma-Aldrich	Cat# M7571
Human recombinant IL-3	Peprotech	Cat# 200-03
Human recombinant IL-6	Peprotech	Cat# 200-06
Recombinant His-tagged human CD38 protein	Sino Biological	Cat# 10818-H08H
Recombinant His-tagged human ENPP1	R&D	Cat# 6136-EN
Bafilomycin A1	Sigma-Aldrich	Cat# B1793
CD38 Inhibitor 78C	Sigma-Aldrich	Cat# 5.38763
β-NAD, ε-NAD	Sigma-Aldrich	Cat# N0632, N2630
Hexadimethrine bromide (Polybrene)	Sigma-Aldrich	Cat# 107689
G418 (Geneticin)	Sigma-Aldrich	Cat# A1720
Snake Venom Phosphodiesterase (SVPDE)	Sigma-Aldrich	Cat# P3243
C_12_FDG	Invitrogen	Cat# D2893

**Critical commercial assays**		

2’3’-cGAMP ELISA Kit	Cayman Chemical	Cat# 501700
M-PER Mammalian Protein Extraction Reagent	ThermoFisher	Cat# 78501
DuoSet ELISA human IFN-β	R&D	Cat# DY814-05
CD138 MicroBeads, human	Miltenyi Biotec	Cat# 130-097-614
APC Annexin V Apoptosis Detection Kit with PI	BioLegend	Cat# 640932
2x SensiFAST Probe Hi-ROX Mix	Meridian Bioscience	Cat# BIO-82020
Lipofectamine 2000	ThermoFisher	Cat# 11668019
His-Tag Labeling Kit RED-tris-NTA 2nd Generation	Nanotemper Technology	Cat# MO-L018

**Experimental models: Cell lines**		

SKO-007(J3)	Provided by Prof. P. Trivedi (Sapienza University of Rome, Italy)	N/A
ARK	Provided by Prof. P. Trivedi (Sapienza University of Rome, Italy)	N/A
ARH-77	ATCC	CRL-1621
THP-1	Provided by Prof. J. Hiscott (Pasteur Institute, Rome, Italy)	TIB-202
293T	ATCC	CRL-3216
NKL	Provided by M. J. Robertson (Indiana University School of Medicine, Indianapolis, USA)	N/A
A549	Provided by Prof. J. Hiscott (Pasteur Institute, Rome, Italy)	CCL-185

**Oligonucleotides**		

sh*CD38* sequence: GCATACCTTTATTGTGATCTA	Sigma-Aldrich	TRCN0000050868
scramble sequence: GCGCGATAGCGCTAATAATTT	Sigma-Aldrich	SHC016

**Recombinant DNA**		

pLKO.1-neo, MISSION custom vector	Sigma-Aldrich	Cat# SHCLND
pVSV-G	Addgene	Cat# 138479
psPAX2	Addgene	Cat# 12260

**Software and algorithms**		

FlowJo v10.8.1	Tree Star	https://www.flowjo.com
ImageJ	NIH	https://imagej.nih.gov/ij/
iBright Analysis Software	Invitrogen	N/A
GraphPad Prism version 8	GraphPad	https://www.graphpad.com/scientificsoftware/prism/
MO.Control software v1.5.3	Nanotemper Technologies	https://shop.nanotempertech.com/en/mocontrol-software-1-license-32
Affinity analysis software v2.2.7	Nanotemper Technologies	https://shop.nanotempertech.com/en/moaffinity-analysis-software-unlimited-licenses-34
Rosetta 3.13	Bender et al. 2016^51^	https://www.rosettacommons.org/
OpenBabel 3.1.0.	O'Boyle et al. 2011^52^	https://github.com/openbabel/openbabel
Biochemical Library (BCL)	Brown et al. 2022^53^	https://github.com/BCLCommons/
GROMACS 2020.4	Van Der Spoel et al. 2015^54^	https://www.gromacs.org/
cpptraj	Roe and Cheatham 2013^55^	https://github.com/Amber-MD/cpptraj
MDAnalysis	Michaud-Agrawal et al. 2011^56^	https://www.mdanalysis.org/
MMPBSA.py	Miller et al. 2012^57^	http://ambermd.org/
POVME 3.0	Wagner et al. 2017^58^	https://github.com/POVME/POVME3
UCSF Chimera 1.14	Pettersen et al. 2004^59^	https://www.cgl.ucsf.edu/chimera/
ChimeraX	Goddard et al. 2018^60^	https://www.cgl.ucsf.edu/chimerax/

**Other**		

BD Horizon Fixable Viability Stain 780	BD Biosciences	Cat# 565388
Zombie Green Fixable Viability Kit	BioLegend	Cat# 423112

### Resource availability

#### Lead contact

Further information and requests for resources and reagents should be directed to and will be fulfilled by the lead contact, Alessandra Soriani (alessandra.soriani@uniroma1.it).

#### Materials availability

Cell lines generated in this study are available upon request to the lead contact.

#### Data and code availability

• Data reported in this paper will be shared by the lead contact upon request
• This paper does not report original code
• Any additional information required to reanalyze the data reported in this paper is available from the lead contact upon request

### Experimental model and study participant details

#### Cell lines

The human multiple myeloma (MM) cell lines SKO-007(J3) and ARK were kindly provided by Prof. P. Trivedi (“Sapienza” University of Rome, Italy), while the cell line ARH-77 was purchased from ATCC. THP-1 and A549 cells were kindly provided by Prof. J. Hiscott and Dr. E. Palermo (Istituto Pasteur Italia). These cell lines, except for A549, were maintained in RPMI-1640 (Life Technologies, Gaithersburg, MD) supplemented with 15% Fetal Calf Serum (FCS, Gibco), 2 mM glutamine and 100 U/mL penicillin 100 μg/mL streptomycin at 37°C and 5% CO_2_. NKL cells were maintained in complete medium enriched with recombinant human IL-2 (200 U/ml; PeproTech, London, UK). The human 293T embryonic kidney cell line was purchased from ATCC and, similarly to A549, was maintained in Dulbecco’ s modified Eagle’s supplemented with 10% FCS, 2 mM L-glutamine, 100 U/ml penicillin, and 100 U/ml streptomycin.

#### Clinical samples

Bone marrow samples from MM patients with different diagnoses were managed at the Department of Cellular Biotechnologies and Hematology, Institute of Hematology (“Sapienza” University of Rome, Italy). Informed consent in accordance with the Declaration of Helsinki was obtained from all patients, and approval was obtained from the Ethics Committee of the “Sapienza” University of Rome (Rif. 3373). BM mononuclear cells (BMMCs) were isolated by Ficoll gradient (Lympholyte, Euroclone); red blood cells were lysed with a buffer composed of 1.5M NH_4_Cl, 100mM NaHCO_3_, and 10mM EDTA.

Bone marrow stromal cells were isolated through plastic adhesion from untreated BMMCs in MEMα medium supplemented with 10% FCS, 2 mM glutamine and 100 U/mL penicillin 100 μg/mL streptomycin at 37°C and 5% CO_2_. In line with the International Society for Cellular Therapy (ISCT) recommendations, a combination of antibodies, including anti-CD45/APC-H7, anti-CD90/PeCy5, anti-CD105/APC, anti-CD146/BV395, anti-CD73/V450 PEA, anti-CD106/PE, was used to phenotypically characterize BMSCs by multiparameteric flow cytometry, as described elsewhere.^27^ The immunophenotype was analysed using a FACS LRSFORTESSA flow cytometer (BD Biosciences, San Jose, CA, USA).

Primary PCs were maintained at 37°C and 5% CO_2_ in complete RPMI medium (or in medium without FCS for ELISA experiments) supplemented with 20 ng/mL human recombinant IL-3 and 2 ng/mL human recombinant IL-6 (PeproTech).

### Method details

#### Isolation of malignant PCs from MM patients

Percentage of CD38/CD138 double-positive cells among BMNCs, representing malignant plasma cells, was determined by flow cytometry. CD138-positive malignant PCs were isolated from BMNCs by using anti-CD138 conjugated magnetic beads (CD138 MicroBeads, human, Miltenyi Biotec) according to the manufacturer’s protocol. A buffer composed of PBS w/o calcium and magnesium, 0.5% Bovine Serum Albumine, 2 mM EDTA was used for the magnetic separation.

#### Drugs and chemicals

Doxorubicin and melphalan were purchased from Sigma Aldrich, bortezomib and carfilzomib from Selleckchem. 2’3’-cGAMP and 2’3’-cG^s^A^s^MP were purchased from Invivogen, CD38 Inhibitor 78C (CAS 1700637-55-3), MK-571 (CAS115103-85-0) and NAD from Sigma-Aldrich. Ultrapure solvents and chemicals for LC-MS analyses were from Romil (Cambridge, UK).

#### Flow cytometry and antibodies

For flow cytometry experiments, cells in PBS w/o Ca and Mg were incubated with antibodies directed against surface proteins for 30 minutes at 4°C. Acquisition was performed on a BD FACSCanto II cytometer.

The following antibodies were used for flow cytometry: anti-CD138/FITC (clone MI15, BD Biosciences); anti-CD38/APC (clone HIT2, BD Biosciences); anti-ENPP1/APC (polyclonal sheep IgG, R&D), polyclonal sheep IgG/APC (R&D). The following antibodies from BD Biosciences were used for validating the immunophenotype of BMSCs: anti-CD45/APCH7 (clone 2D1), anti-CD105/APC (clone 266), anti-CD90/PeCy5 (clone 5E10), anti-CD146/BV395 (clone P1H12), anti-CD106/PE (clone 51-10C9) and anti-CD73/V450 (clone AD2).

For the exclusion of dead cells, the following dyes were used: BD Horizon Fixable Viability Stain 780 (APC-H7); Zombie Green Fixable Viability Kit (BioLegend).

For the quantification of Senescence-associated β-galactosidase activity, the substrate C_12_FDG (5-Dodecanoylaminofluorescein Di-β-D-Galactopyranoside) was used for flow cytometric experiments. Cells were incubated 1h at 37°C and 5% CO_2_ with bafilomycin A1 100 nM (Sigma) in culture medium to induce lysosomal alkalinization at pH 6 and then for 1h with C_12_FDG 33 μM (Invitrogen). The C_12_FDG signal was measured on the FITC detector, and β-galactosidase activity was estimated using the median fluorescence intensity (MFI) of the population.

For the detection of apoptotic cells, APC Annexin V Apoptosis Detection kit with PI (propidium iodide) (BioLegend) was used according to the manufacturer’s protocol. Early apoptotic cells are Annexin V-positive PI-negative, whereas late apoptotic and necrotic cells are double-positive.

All flow cytometry data were analyzed using FlowJo 10.8.1 Software (BD Biosciences).

#### SDS-PAGE and western blot

For Western-Blot analysis, MM cell lines and purified patient-derived myeloma cells were pelleted, washed once with cold PBS, resuspended in lysis buffer [2.5 mM EDTA, 2.5 mM EGTA, 50 mM Tris-HCl pH 7.5, 150 mM NaCl, 1.5 mM MgCl_2_, 1.0% Triton-X-100, 5 mM NaF, 1 mM Na_3_VO_4_, 1 mM PMSF, Protease Inhibitor Cocktail 1X (Sigma-Aldrich, St. Louis, Missouri, USA), Phosphatase Inhibitor Cocktail 3 1X (Sigma-Aldrich)] and then incubated for 30 minutes on ice. The lysates were centrifuged at 16,000 g for 20 min at 4°C and the supernatants were collected as whole-cell extract. Protein concentration was determined through Bradford Protein Assay (Bio-Rad). 10 to 65 μg of cell extracts were run on 10% denaturing SDS-polyacrylamide gels. Proteins were then electroblotted onto Amersham Protran nitrocellulose membranes (GE Healthcare Life Science), stained with Ponceau to verify that similar amounts of proteins had been loaded in each lane, and blocked with 5% BSA or non-fat dry milk in TBST buffer for 1h. Immunoreactive bands were visualized using horseradish peroxidase-linked/coupled donkey anti-rabbit (NA934V) or sheep anti-mouse (NA931V) IgG (Amersham, GE Healthcare Life Science) and the ECL substrate WESTAR ηC ULTRA 2.0 (Cyanagen).

Primary antibodies used: rabbit anti-human cGAS (clone D1D3G, Cell Signaling Technology), rabbit anti-human PI3 Kinase p85, N-SH2 domain (ABS233, Merck Millipore).

Image acquisition was performed on an iBright CL1500 (Invitrogen). Densitometric analysis was performed on ImageJ 1.53k (NIH, USA). Target protein levels were referred to p85, chosen to normalize protein expression.

#### ELISA for 2’3’-cGAMP and IFN-β detection

To quantify cGAMP, a commercial competitive ELISA kit was used (2’3’-cGAMP ELISA Kit, Cayman Chemical) according to the manufacturer’s protocol. The conditioned medium was, for every cell line, obtained with RPMI-1640 in absence of FCS, collected after 24 hours, and tested undiluted in duplicate or triplicate. Cell lysates were obtained from 0.5 · 10^6^ cells in 50 μL of lysis buffer (M-PER Mammalian Protein Extraction Reagent, ThermoFisher) and tested undiluted in duplicate. Calculations were performed with the use of a ready-made spreadsheet provided by the manufacturer (available at www.caymanchem.com/analysis/elisa).

IFN-β secreted by BMSCs in the undiluted CM was quantified using a DuoSet ELISA (R&D, DY814-05) according to the manufacturer’s protocol.

#### Reverse-transcription (RT) and Real time PCR

Total RNA was extracted with Trizol (Invitrogen) and chloroform/ethanol precipitation or with Total RNA mini kit (Geneaid). RNA concentration was measured with a Nanodrop spectrophotometer (ThermoFisher Scientific). When used for real time amplification of *IFN-β*, RNA was treated with DNase I (DNase I Amplification Grade, Sigma-Aldrich) before RT-PCR.

Total RNA (300 ng to 1 μg) was used for cDNA first- strand synthesis using oligo-dT, dNTPs and M-MLV Reverse Transcriptase with its 5X buffer (Promega, Madison, WI) in a 25 μL reaction volume.

Real time PCR was performed using TaqMan probes (Master Mix: 2x SensiFAST Probe Hi-ROX Mix, Meridian Bioscience): *CD38*: Hs01120071_m1, *cGAS*: 00403553_m1, *CXCL10*: Hs00171042_m1, *IFN-β:* Hs01077958_s1, *IL-15*: Hs00174106_m1, *GAPDH*: Hs02758991_g1.Where indicated, genes were amplified using a TaqMan Array 96-well FAST Plate, Custom format 16 (Applied Biosystems). The Probes used were the following:

*GUSB*: Hs99999909_m1; *IFNA1*: Hs03044218_g1; *IFNA2*: Hs00265051_s1; *IFNA4*: Hs01681284_sH; *IFNB1*: Hs01077958_s1; *IFNE*: Hs00703565_s1; *IFNW1*: Hs00958789_s1; *IFNL1*: Hs00601677_g1; *IFI6*: Hs00242571_m1; *IFIH1*: Hs00223420_m1; *IFIT1*: Hs03027069_s1; *IFIT2*: Hs01584837_s1; *IFIT3*: Hs01922752_s1; *IFITM1*: Hs00705137_s1; *IRF7*: Hs01014809_g1; *ISG15*: Hs01921425_s1; *ISG20*: Hs00158122_m1; *IFI27*: Hs01086373_g1; *IFI44L*: Hs00915292_m1; *RSAD2*: Hs00369813_m1; *DDX58* (RIG-I): Hs01061436_m1; *TGFB1*: Hs00998133_m1; *IL10*: Hs00961622_m1; *IL15*: Hs01003716_m1; *IL6*: Hs00174131_m1; *IL1B*: Hs01555410_m1; *CCL5*: Hs00982282; *CXCL9*: Hs00171065_m1; *CXCL10*: Hs00171042_m1; *CXCL11*: Hs00171138_m1; *IFNG*: Hs00989291_m1; *ENPP1*: Hs01054040_m1; *CD73*: Hs00159686_m1.

Results were analyzed by subtracting, for each condition, the average Ct of the samples to the average Ct of housekeeping genes (GAPDH and GUSB) and were reported as fold change in respect to a reference condition, calculated with the method of 2^-ΔΔCt^.

#### Lentiviral transduction

Plasmids carrying shRNA construct (pLKO.1-neo, MISSION custom vectors) were purchased from Sigma Aldrich: shCD38 sequence: GCATACCTTTATTGTGATCTA (Clone ID: TRCN0000050868), scramble sequence: GCGCGATAGCGCTAATAATTT (Clone ID: SHC016).

For lentiviral particles production, viral vectors were cotransfected with the packaging vectors pVSVG and psPAX2 (Addgene) into 293T cells using Lipofectamine 2000 (Life Technologies). After transfection, cells were placed in a fresh medium. After additional 48h culture, virus-containing supernatants were harvested, filtered (0.22 μm) and used immediately for infections. Infections were performed on 1.0 × 10^6^ ARK cells per mL of viral supernatant containing polybrene (8 μg/ml) (Hexadimethrine bromide — Sigma-Aldrich) for 1 h. Selection of cells carrying the shRNA construct was carried out in 0.5 mg/mL G418 (Sigma-Aldrich).

#### Fluorescence quenching binding measurements

Fluorescence quenching measurements were performed a on Cary Varian Eclipse fluorescence spectrophotometer equipped with a Peltier thermostatted system using a 10-mm path length quartz cell with a Teflon stopper (quartz cuvette of 1 cm path length).

The recombinant human His-tagged CD38 protein (rhCD38, Sino Biological) at the fixed concentration of 1 μM (1 pmol/μL = 400 pmol in total) was titrated in 25 mM phosphate pH 7.5, 50mM NaCl, with increasing concentrations of ligands 2’3’-cGAMP, 2’3’-cG^s^A^s^MP and β-NAD dissolved in the same buffer. The Trp fluorescence spectra of rhCD38 in the absence and presence of ligands were acquired at 25.0°C, using an excitation wavelength of 295.0 nm to avoid interference with tyrosine residues, and a fluorescence emission wavelength ranging from 300 nm to 500 nm. The excitation and emission slits were set at 5 nm; the scan rate was 120 nm/min; the data interval was 1.0 nm; the averaging time was set at 0.5 s. The samples were allowed to equilibrate for 1 min prior to measurement. The emission spectra of the ligand solutions were acquired under the same conditions, using solutions up to 205 μM, in order to exclude interference from ligands intrinsic fluorescence. The fluorescence values recorded at 345 nm were extracted, normalized to the intrinsic fluorescence of rhCD38, and then plotted as an average trace against the ligand concentrations. The value of the dissociation constant (K_D_) for different ligands was calculated by fitting fluorescence quenching data using a non-linear regression analysis on GraphPad Prism (Graph Pad Software).

#### Microscale thermophoresis binding analysis of cGAMP to CD38 and ENPP1

Binding measurements were carried out as reported previously^61^ with a Monolith NT.115 device using MST Premium capillaries (Nanotemper Technologies GmbH, Munich, Germany). The MO.Control software v1.5.3 from Nanotemper Technologies was used for data acquisition and manipulation. For the assays, rhCD38 and rhENPP1 were labeled using the His-Tag labeling kit RED-tris-NTA 2^nd^ Generation (Nanotemper Technologies GmbH, Munich, Germany).^62^^,^^63^ Labeling was performed according to the protocol provided by the manufacturer using proteins at 100 nM in an optimized labelling buffer (10 mM Hepes pH 7.2). For the binding assays, rhCD38 and rhENPP1 were used at a final concentration of 50 nM. cGAMP was used at concentrations ranging between 200 μM and 6 nM (binding to rhCD38) or 100 μM and 3 nM (binding to rhENPP1). The final reaction volume was 10 μL. Two independent experiments were performed, reporting data as average ± SE. Affinity analysis software v2.2.7 (Nanotemper Technologies GmbH, Munich, Germany) was used to estimate the K_D_ applying the model for binding interactions with a predicted 1:1 stoichiometry. Binding data were imported and analyzed with GraphPad Prism. The dissociation constant, K_D_, is obtained by fitting a non-linear dose-response curve to a plot of F_norm_ vs ligand concentration. F_norm_ relates to the fluorescence values prior (F_0_) to and after (F_1_) IR laser activation.

#### Supplemental biochemical methods

##### Testing cGAMP degradation by CD38 using LC-MS

cGAMP at 200 nM in 10 mM PBS pH 7.0 was incubated with rhCD38 (4 nM, enzyme:substrate ratio 1:50) at 37°C for at least 180 minutes and analyzed by LC-MS at the following time points: 0, 30, 60, 90, 120, 180 min. 100 μL were removed at the indicated time points, frozen in dry ice and analyzed as described below (5 μL, 1 pmole). rhCD38 alone was analyzed in the same conditions. At least 2 independent experiments were performed, and data represented as mean ± SEM.

Samples were analysed with a ThermoFisher LC-MS system equipped with a Ultimate 3000 HPLC comprising a binary pump, an automated autosampler, a multi-wavelength Diode Array detector, and an Orbitrap high-resolution mass spectrometer (Q-Exactive Plus, max resolution 280,000). An Acclaim™ PepMap™ 100 C18 HPLC Column 100 mm x 1 mm ID, 3 μm (ThermoFisher, Milano) was used for all analyses. The column was operated at a flow rate of 0.15 mL/min and maintained at 40°C in all analyses. Solvent A was H_2_O added with 0.05% formic acid (FA); solvent B was acetonitrile added with 0.05% FA. The gradient used in all analyses was: 1% solvent B for 2 minutes; from 1% to 50% solvent B in 13 minutes. The column was washed 5 minutes with 90% solvent B and re-equilibrated to 1% solvent B for 6 minutes. The absorbance trace at 260 nm was recorded between 0 and 28 minutes. The Q-Exactive MS was operated in the positive ion mode between 100 and 1100 m/z at a resolution of 35,000 in full scan mode. The AGC target was set at 1x10^6^, the inject time was 20 ms (1 microscan). In the data dependent mode (DDA) the resolution was set at 17,500, the AGC target was 1x10^6^, the inject time was 100 ms, the isolation window was set to 2.0 m/z, the inject time was maintained at 100 ms. The three most intense peaks were selected for fragmentation at a stepped collision energy of 20. The source was maintained at 3.50 kVolts and 350°C. The desolvating N2 gas was kept at 22 L/min and at 50°C. The S-lens RF level was set at 50. Mass data were collected between minute 1 and minute 20 in all analyses. cGAMP was detected as doubly charged ion at m/z 338.057 and quantified by integrating the LC peaks obtained extracting the ions at m/z 338.060 ± 0.010 ([M+2H]^2+^). A 11 points smoothing algorithm was applied to all runs.

Mass peaks corresponding to linearized cGAMP at m/z 693.118 ± 0.010 ([M+H]+), AMP at m/z 348.071 ± 0.010 ([M+H]+), GMP at m/z 364.066 ± 0.010 ([M+H]+), adenosine at m/z 268.104 ± 0.010 ([M+H]+), adenine at m/z 152.057 ± 0.010 ([M+H]+), guanosine at m/z 284.099 ± 0.010 ([M+H]+), and guanine at m/z 136.062 ± 0.010 ([M+H]+) were also extracted to check for the presence of these possible cGAMP degradation products (Table S1).

##### Testing cGAMP degradation by recombinant ENPP1

Degradation of cGAMP by ENPP1 was tested incubating the dinucleotide at 200 nM and ENPP1 at 4 nM (enzyme:substrate ratio 1:50) in 10 mM HEPES pH 7.0, 1 mM CaCl2. 5 μL samples were analyzed with the method reported above at 0, 60 and 90 minutes. cGAMP was detected as a doubly charged ion at m/z 338.0565 and quantified by integrating the LC peaks obtained extracting ions at m/z m/z 338.060 ± 0.010 ([M+2H]2+). AMP and GMP were revealed by extracting the [M+H]^+^ ions at m/z 364.066 ± 0.010 (GMP, calculated m/z 364.0658; experimental m/z 364.0656) and m/z 348.070 ± 0.010 (AMP, calculated m/z 348.0708; experimental m/z 348.0705) (Table S2).

##### Testing the hydrolase activity of rhCD38 and its inhibition by cGAMP

The hydrolase activity of CD38 was assessed by fluorescence-based assays using rhCD38 and the synthetic substrates 1, N6-Ethenonicotinamide adenine dinucleotide (ε-NAD, Sigma-Aldrich). Assays were performed in 96-well black polyethylene plates. Reaction mixtures (0.2 mL) containing rhCD38 (0.5 nM) and the substrate in 50 mM MES buffer pH 6.0 were prepared in triplicate wells. Kinetics measurements were recorded on a Synergy multi-wavelength plate reader (Biotek, Winooski, VT, USA), equipped with a thermostated chamber. Hydrolysis of ε-NAD by rhCD38 was estimated adding the substrate at concentrations ranging from 2.5 μM to 75 μM and monitoring the emission at 410 nm (excitation 310 nm) for 8 min at 37°C. The ability of cGAMP to inhibit the enzyme hydrolase activity was tested pre-incubating rhCD38 with the dinucleotide for 10 min at 37°C at concentrations ranging from 1.25 μM to 40 μM, before adding ε-NAD at a 25 μM. As positive control the commercial CD38 inhibitor 78c was used at 50 nM, 100 nM and 200 nM.

Data were processed extrapolating the slope of the saturating curves in each well by linear regression analysis. The K_m_ value for ε-NAD was extrapolated by fitting the data with GraphPad Prism (GraphPad Prism version 5.00, GraphPad Software, San Diego California USA) plotting the slope values versus nucleotide concentration and fitting the data using a nonlinear curve-fitting method implemented in the software. Enzymatic activity was reported as U/mL as a function of substrate concentration, where U/mL units were calculated as the ratio between slope and the coefficient of molar extinction of the enzyme normalized for the reaction volume. The percentage of inhibition of cGAMP toward the CD38 hydrolase activity was determined by comparing the activity measured in the inhibition assays with those resulting from control experiments (assumed as 100%).

#### Computational methods

##### Protein preparation

Before the start of the computational modeling, the unbound crystal structure of CD38 that showed the highest data quality, coverage and best resolution according to PDBe-KB^64^(PDB ID: 4CMH) was downloaded and prepared for use in Rosetta,^51^^,^^65^ first isolating chain A from the .pdb file with the “clean_pdb.py” Python script within the Rosetta package and then performing an all-atom coordinate-constrained relaxation, following the protocol from Nívon et al.^66^ The latter uses cycles of minimization with combined backbone/sidechain restraints that is Pareto-optimal with respect to RMSD to the native structure and energetic strain reduction. The structure with the lowest total score out of ten generated structures was used for further work (i.e., RosettaLigand docking run and unbound CD38 MD simulations).

##### Ligand preparation

The initial structure of the 2’3’-cGAMP molecule was downloaded from the Chemical Entities of Biological Interest (ChEBI) database (https://www.ebi.ac.uk/chebi)^67^ in the 1D simplified molecular-input line-entry system (SMILES) string file format. OpenBabel 3.1.0 version^52^ was used to obtain a 2D representation of the atomic coordinates. Subsequently, the Gen3D module^68^ of the Open Babel package^52^ was used to generate and optimize the geometry of a 3D model for the 2’3’-cGAMP compound which was saved as a 3D-structure-data file format (SDF). Protonation and tautomeric states for 2’3’-cGAMP were assigned with Open Babel^52^ assuming a pH of 7.4. The structure was then minimized under the Generalized Amber force field (GAFF)^69^ performing 2,500 steps of minimization using the conjugate gradient algorithm. Following the procedure in ref.^70^ the “molecule:Filter” application in the Biochemical Library (BCL) software^53^ was used to clean the obtained SDF file and check for incorrect bond order assignments, undesired protonation states/formal charges that cannot be easily and automatically corrected.

##### Molecular Docking

RosettaLigand and RosettaScripts applications^71^^,^^72^^,^^73^ from Rosetta macromolecular modeling suite version 3.12^51^^,^^74^ was used to dock 2’3’-cGAMP to CD38. The processed (see above) crystal structure of CD38 in the unbound state exhibiting the lowest Rosetta energy score was chosen for docking of 2’3’-cGAMP. Docking was comprised of three stages, that progressed from low-resolution conformational sampling and scoring to full-atom optimization using all-atom energy function. In the first low-resolution stage, 2’3’-cGAMP molecule was initially placed roughly in the center of the space defined by the catalytic site residues. 2’3’-cGAMP conformers were generated with the BCL conformer generator (BCL::Conf) using 10,000 iterations to create a maximum of 250 conformers.^75^ The molfiletoparams.py Python script within the Rosetta package was used to assign atomic chemical properties and generate parameter definitions for the 2’3’-cGAMP molecule. All conformers were then randomly rotated as a rigid body and scored for shape compatibility with the CD38 catalytic site. During the low-resolution docking phase, each ligand is allowed to explore the binding site in a 10.0 Å radius. Rigid body transformation is combined with ligand conformation swaps for 1000 (default 500) cycles of Monte Carlo Metropolis optimization. The best-scoring models were filtered by RMSD to eliminate near-duplicates and one of the remaining models was selected at random to continue to the next stage. The second (high-resolution) stage employed the Monte Carlo minimization protocol in which the ligand position and orientation were randomly perturbed by a small deviation (0.1 Å and 3°). During the high-resolution docking phase, 24 (default 6) cycles of side-chain rotamer and ligand conformer sampling were performed. CD38 catalytic site residue side-chains were repacked using a rotamer library; the ligand position, orientation, torsions, and protein side-chain torsions were simultaneously optimized using quasi-Newton minimization and the end result was accepted or rejected based on the Metropolis criterion. The side-chain rotamers were searched simultaneously during “full repack” cycles and one at a time in the “rotamer trials” cycles. The full repack made ∼106 random rotamer substitutions at random positions and accepted or rejected each based on the Metropolis criterion. Rotamer trials chose the single best rotamer at a random position in the context of the current state of the rest of the system, with the positions visited once each in random order. The ligand was treated as a single residue and its input conformers served as rotamers during this stage. The third and final stage was a more stringent gradient-based minimization of the ligand position, orientation, and torsions and the channel torsion angles for both side-chains and backbone. Scoring uses the same Rosetta energy function, but with a hard-repulsive van der Waals potential; this creates a more rugged energy landscape that is less suited to the search stage (above) but is better at discriminating native from non-native binding modes.^51^^,^^73^

A total of 20,000 models were generated for each docking trial parallelized using the Message Passing Interface (MPI). To select the best docking model, these models were first screened with total energy score (Rosetta energy term name: total_score). Top 1,000 models with lowest total energy score were selected. They were further scored with the binding energy between 2’3’-cGAMP and CD38 (Rosetta energy term name: interface_delta_X). Binding energy was calculated as the difference in total energy between the 2’3’-cGAMP bounded state and the corresponding unbound state models. Top 10 models with lowest binding energy (interface_delta_X) were identified as the candidates. These models exhibited excellent structural convergence (RMSD <1.5 Å, in particular 9 out of 10 models exhibited a RMSD in the range of 0-to-0.13 Å, while 1 out of 10 models exhibited a RMSD of 1.45 Å). Indeed, the convergence of the docking run was assessed in energetic and structural terms, evaluating the “total_score vs interface_delta_X” and “ligand RMSD vs total_score”, respectively. The “total_score vs interface_delta_X” showed the sampling of highly stable complexes, characterized by both high overall stability and strong binding energy. To quantify the docking results for structural convergence, the binding energy value for 20,000 docking models were plotted against its unsuperimposed ligand root-mean-squared deviation (ligand RMSD). The unsuperimposed ligand RMSD was computed for each of the 20,000 models using the lowest interface scored structure as the reference pose. The resulting collection of data was then used to calculate the Pnear metric, defined by the following expression:

(Equation 1) $$P_{near}=\frac{\sum_{i=1}^{n}e^{-\left({RMSD}_{i}^{2}/\lambda^{2}\right)}e^{-\left(E_{i}/kBT\right)}}{\sum_{j=1}^{n}e^{-\left(E_{i}/kBT\right)}}$$

where λ = 1.5 and kBT = 0.62.

Pnear was used to quantify a score function’s ability to recapture the “funnel-likeness” of a RMSD vs. energy plot.^70^ The pnear calculation (Equation 1) is typically used to determine folding propensity where a value of 1 suggests a protein exists solely in the native state (0 Å), whereas a pnear of 0 suggests a protein will never exist in the native state. In a docking study, the “Pnear” metric quantify a score function’s ability to recapture the experimentally determined binding pose or, as in this case, the “funnel-likeness” of a RMSD vs. energy plot.

To quantitatively analyze the docking results, binding energy was decomposed as van der Waals (VDW) energy, hydrogen bond energy (if_X_hbond_sc) and electrostatic interaction (if_X_fa_pair). VDW was calculated as the sum of attractive and repulsive components (if_X_fa_atr and if_X_fa_rep, respectively).

The total VDW and electrostatic energies ranged from 0 to −6 Rosetta Energy Units (R.E.U.), while hydrogen bond energy reached a maximum score of -2.

To reveal the spatial distribution of binding interaction between 2’3’-cGAMP and CD38, VDW and hydrogen bond energies were further mapped on a per residue basis to CD38 by Rosetta’s residue_energy_breakdown utility. Average values of VDW energy, hydrogen bond energy and electrostatic interaction were calculated based on the top 10 models with the lowest binding energy. The top scoring docked model is used for visualizing protein – ligand interface in UCSF Chimera 1.14.^59^

##### Molecular Dynamics (MD) simulation

###### System setup

####### unbound CD38

the enzyme structure corresponding to the lowest total score relaxed structure was used (see “protein preparation” section). Protein protonation states were assigned at pH 7.4 based on an optimal hydrogen bonding conformation.^76^

####### bound CD38

the lowest interface scored structure of the CD38 enzyme in complex with 2’3’-cGAMP obtained from the docking run was subjected to an all-atom coordinate-constrained relaxation.^66^

##### MD simulations

We performed all-atoms classical molecular dynamics (MD) simulations of unbound and bound CD38 systems. The MD simulations were carried out using GROMACS 2020.4 program package.^76^ Proteins were modeled with the AMBERff14SB force field,^77^ while 2’3’-cGAMP ligand was parameterized using GAFF force field^69^ and AM1-BCC^78^ partial atomic charges by ACPYPE tool.^79^

Each system was solvated with the explicit TIP3P^80^ water model in a dodecahedral box with periodic boundary conditions and a minimum distance between the solute and the box of 10 Å; its net charge was neutralized with the required number of randomly placed Na+ ions.

All bonds involving hydrogen atoms were constrained using the LINCS algorithm.^81^ The SETTLE algorithm was used for water.^82^ The leapfrog algorithm was used to numerically integrate the equation of motions with a time step of 2 fs. A cutoff of 1.2 nm was used for short-range electrostatic and Lennard–Jones interactions. Long-range electrostatic interactions were calculated by particle-mesh Ewald^83^ summation with a fourth-order interpolation and a grid spacing of 0.16 nm. The solute and solvent were coupled separately to a temperature bath using velocity-rescaling thermostat^84^ with a relaxation time of 0.1 ps. The pressure coupling was fixed at 1 bar with a relaxation time of 2 ps and isothermal compressibility of 4.5 × 10−5 bar−1 using the Berendsen weak-coupling algorithm^85^ and the Parrinello–Rahman algorithm^86^ for equilibration and production runs, respectively. Unbound and bound systems were carefully equilibrated using an extensive multistep equilibration protocol, as described by Wallnoefer et el.^87^ In detail, the following equilibration protocol was applied: minimization of hydrogen atoms and water molecules with 1000 steps of steepest descent followed by 1000 steps of conjugate gradient;^64^ 200 ps of NVT simulation increasing the temperature from 100 to 300 K with fixed heavy atoms of the protein and ligand;^51^ 200 ps of NPT simulation^65^ (pressure regulation with the Berendsen weak-coupling algorithm^77^) to adjust the box size with fixed heavy atoms of the protein and ligand; 100 ps of NVT simulation to cool the system to 100 K with fixed heavy atoms of the protein and ligand;^66^ nine minimizations with decreasing positional restraints on the protein and ligand heavy atoms to remove potential steric clashes or other structural problems in the protein (force constants used: 1000, 500, 100, 50, 20, 10, 5, 2, and 0 kJ mol−1 nm−2);^67^ and 100 ps of NVT simulation increasing the temperature from 100 to 300 K.^52^

After equilibration, in order to extensively sample the conformational space, six independent unrestrained MD production runs of 1 μs each system were carried out in the NPT ensemble. This resulted in a total sampling time of 3 x 1 μs for unbound and 3 x 1 μs for bound states. Within this simulation time, total energy, 1D and 2D RMSD of the protein’s backbone atoms converged; therefore, the systems were considered equilibrated and suitable for statistical analysis.

Initial random velocities were sampled from the Maxwell-Boltzmann distribution for each repeat. Coordinates from production trajectories were saved every 10 ps.

Final values in the subsequent analyses are reported by averaging the results from the three replicate simulations of unbound and bound systems separately.

##### Simulations analyses

MD trajectories have been analyzed using GROMACS 2020.4, cpptraj and MDanalysis packages.^55^^,^^56^^,^^76^ RMSD analyses have been performed considering the backbone atoms of CD38 protein residues, while RMSF has been calculated considering only the α-carbons of the protein. For the 2’3’-cGAMP molecule, RMSD and RMSF were calculated on the heavy atoms. As compared to the time-series plot of RMSD with respect to a single reference structure (1D-RMSD), a better RMSD-based convergence measure is the all-to-all RMSD plot (also known as pairwise RMSD or 2D-RMSD plot); taking the RMSD of each snapshot in the trajectory with respect to all others in order to use RMSD for identifying very similar structures. By definition, all such plots have values of zero along the diagonal (as this represents the RMSD of a structure to itself). Blocks of low RMSD values along the diagonal indicate similar structures, suggesting the occupation of a given state. Blocks of low RMSD values off the diagonal indicate that the trajectory is revisiting previously sampled states, a necessary condition for good statistics.

Ligand RMSD indicates how stable the ligand is with respect to the protein and its binding pocket or respect to its starting binding mode. To evaluate the magnitude of the displacements and reorientations of the 2’3’-cGAMP molecule relative to the protein during the simulation, the protein-ligand complex is first aligned on the protein backbone of the reference and then the RMSD of the ligand heavy atoms is measured. If the values observed are significantly larger than the RMSD of the protein, then it is likely that the ligand has diffused away from its initial binding site.

In order to assess the stability of the 2’3’-cGAMP binding mode, the internal fluctuations of the ligand atoms were measured. 2’3’-cGAMP was aligned just on its reference conformation and RMSD was measured (Figure S4E).

Time evolution of the radius of gyration (Rg) of the α-carbons of the CD38 protein residues in the unbound and bound systems have been also analysed. 1D RMSD, RMSF and Rg were calculated using the GROMACS analysis tools,^76^ while 2D-RMSD was calculated using cpptraj analysis program.^55^

##### Clustering analysis

The clustering analysis was performed separately on the combined 3 μs trajectories of unbound and bound states. The DBSCAN (Density-Based Spatial Clustering of Applications with Noise) clustering algorithm^88^ was used as implemented in the cpptraj module.^55^ Clustering was performed such that the minimum distance between points required for forming a cluster was 1.0 Å and the minimum number of points required for forming a cluster was 42.

##### Binding free energy calculations

Protein-ligand MM/GBSA binding free energy and per-residue decomposition were computed with the mmpbsa.py^89^ module of AmberTools20.^90^ gmx-MMPBSA (v1.4.3)^91^ was used to convert the GROMACS trajectories into AMBER format with ParmEd, and subsequently used mmpbsa.py. The concatenated 3 μs bound trajectory was stripped of water and ions. Energies were computed with a surface tension of 0.0072 kcal/ mol/Å2. The non-polar contribution to the solvation free energy was approximated using the LCPO method.^92^ A single trajectory MM/GBSA protocol was adopted to calculate the binding free energy differences, neglecting the solute entropic contribution.

The enthalpic and solvation free energy contributions were computed using 300,000 equally spaced frames spanning 3 μs bound trajectories. All calculations were completed from three independent trajectories and averaged. Since the ΔGbinding was calculated by omitting the entropic term it is referred to as relative binding energy.

In order to obtain a detailed energetic investigation of CD38 – 2’3’-cGAMP complex formation, per-residue decomposition studies of relative binding energy were carried out using MM/GBSA method. The binding energy of residues located within a 5 Å radius from the ligand was decomposed to investigate the partial energy contributions. Only residues contributing with more than kT (∼0.6 kcal/mol at 300 K) are reported.

##### Catalytic site volume calculation

In order to calculate the fluctuation of the active site volume throughout MD trajectories, POcket Volume MEasurer (POVME) version 3.0^58^ used with the default parameters and defining the catalytic site region as the pocket. The software creates a grid of points that are 0.1 Å apart and then finds the set of grid points that are nearest to ligand atoms. Starting from this set of points, it finds grid points up to 3 Å away in all directions. It examines all points that were generated above and removes grid points that are closer to a protein atom than the distance van den Waals radius of that protein atom + 1.09 Å (Hydrogen bond length). With these points removed, it calculates the volume of the set of remaining grid points. Two .pdb files, containing 10,000 frames each, were extracted at regular time intervals from the trajectory files of a 3 μs unbound and a 3 μs bound simulations, respectively. The .pdb files so obtained were used as the input file for POVME 3.0.

Inclusion spheres were defined to encompass the binding site, and seed spheres were selected to include the minimal definition of the pocket, which were placed roughly at the center of the ligand position in the binding pocket. To allow for comparison across unbound and bound states, the same POVME spheres were used for each system and POVME’s convex hull option was turned off. Finally, the results of all frames are averaged, and the standard deviation is calculated.

##### Protein – Ligand interactions

For hydrogen bond interactions between CD38 and 2’3’-cGAMP, the default cpptraj distance (maximum distance from acceptor to donor heavy atom equal to 3.0 Å) and donor-hydrogen-acceptor angle cut-off (135°) were considered.

The salt bridges interactions analysis has been performed through the cpptraj package, considering only the charged residues R127, K190 and E226, imposing a cut-off distance of 4 Å from the protonated amines or deprotonated oxygen of the 2’3’-cGAMP ligand.

Nonspecific hydrophobic contacts were identified when the nonpolar atoms of an amino acid residue fell within 4 Å from a ligand’s nonpolar carbon.

π - stacking interactions were characterized by face-to-face or face-to-edge stacking between W125, W176, W189 amino acid residue of CD38 and adenosine, guanosine, guanosine-linked ribose (ribG), adenosine-linked ribose (ribA) moieties of 2’3’-cGAMP.

We defined face-to-face stacking using a combination of three criteria: (i) a minimum distance between any pair of heavy atoms in the two ring systems < 4 Å, (ii) a distance between the center of mass of each ring system of <5 Å, and (iii) a vector angle between the normals to the planes of the two rings between 0 and 45° or between 135 and 180°.

In addition, edge-to-face stacking interactions were defined according to the following criteria: (i) a minimum distance between any pair of heavy atoms in the two rings < 4 Å and (ii) a vector angle between the normals to the planes of the two rings between 45 and 135°. The vector angle has been calculated taking the dot product between the normal vectors of the two rings under consideration. This has been carried out using the vector and vectormath commands of cpptraj package.

For the hydrogen bonds and salt bridges analyses, given the high number of interactions observed, only the interactions with a persistence higher than 25% of the simulation time have been reported to limit the description to the most relevant ones. For the π - stacking interactions, the contacts observed for more than 20% of the simulation time are reported.

Protein – Ligand interactions were computed using 300,000 equally spaced frames spanning 3 μs of the bound trajectories. Finally, raw data have been parsed and plotted via scripts written in Python using the matplotlib and seaborn libraries for plotting, and pandas, numpy and scipy for data handling and statistics. Protein-ligand figures have been produced using UCSF Chimera 1.14 [17] and ChimeraX.^60^

##### Principal component analysis

Principal component analysis (PCA) was performed in order to extract the principal modes of motion from the trajectories of unbound and bound systems.^37^ The two trajectories (3 μs each) were concatenated, and the overall translational and rotational motion was removed by least-squares fitting the coordinate data to the first frame. The covariance matrices of positional fluctuations (Cα only) were built and diagonalized, providing a set of eigenvectors ordered according to the corresponding eigenvalues. Construction and diagonalization of the covariance matrix and 2D projections of eigenvectors was performed using the “gmx covar” and the “gmx anaeig” modules of GROMACS, respectively.

A majority of the variance in the molecular motions could be explained by the first two principal components (∼ 80%), with 68.4 % of the variance explained by the first eigenvector alone (Figure 4D). Consequently, only the first two principal components were used for subsequent analysis (Figures 4D and 4E). Finally, the 2D projections of eigenvectors were investigated by Gaussian kernel density estimate (KDE) to create a probability distribution function in subspaces spanned by principal components 1 and 2 (PC1 vs. PC2). Unbound and bound were projected onto the essential subspace generated by the 6 μs concatenated MD trajectories.

The approach that combined a dimension reduction step (PCA) with subsequent clustering (KDE) to analyze MD trajectories data were shown to be capable of reducing the noise and to generate more compact and well separated clusters of conformations.^74^ Thus, for each state (i.e.: unbound and bound), the KDE plots allowed the identification of the higher populated conformational basins.

In order to visualize the motions represented by the eigenvectors, the structures from the ensembles can be projected onto each eigenvector of interest and transformed back into Cartesian coordinates. The two extreme projections along the eigenvector (e.g., the most open and most closed states of a “inter-domain clamping” motion) can then be interpolated to create an animation (Video S1).

### Quantification and statistical analysis

When testing replicate experiments with cell lines, paired Student’s T test (two conditions), one-way ANOVA with matched data followed by Tukey’s test for multiple comparisons (three or more conditions) and two-way ANOVA followed by Bonferroni correction for multiple comparisons (more than two condition across two or more groups) were used. Experiments involving samples derived from different patients were analyzed with one sample T test (for mRNA fold increase) or one-way ANOVA with unmatched data followed by Tukey’s test for multiple comparisons. The corresponding test is reported for each experiment in the figure legends. Error bars represent SEM in all experiments. All statistical tests and corresponding p values calculations were performed using GraphPad Prism v.8.
