# Supplementary material for: CD38 restrains the activity of extracellular cGAMP in a model of multiple myeloma
Source: iScience. 2024 Apr 25;27(5):109814. doi: 10.1016/j.isci.2024.109814 (PMC11091702; doi:10.1016/j.isci.2024.109814)
Supplement: Document S1. Figures S1–S9 and Tables S1 and S2 [file mmc1.pdf]

iScience, Volume *27*

Supplemental information

CD38 restrains the activity of extracellular cGAMP in a model of multiple myeloma

Lorenzo Cuollo, Samuele Di Cristofano, Annamaria Sandomenico, Emanuela Iaccarino, Angela Oliver, Alessandra Zingoni, Marco Cippitelli, Cinzia Fionda, Sara Petillo, Andrea Kosta, Valentina Tassinari, Maria Teresa Petrucci, Francesca Fazio, Menotti Ruvo, Angela Santoni, Domenico Raimondo, and Alessandra Soriani

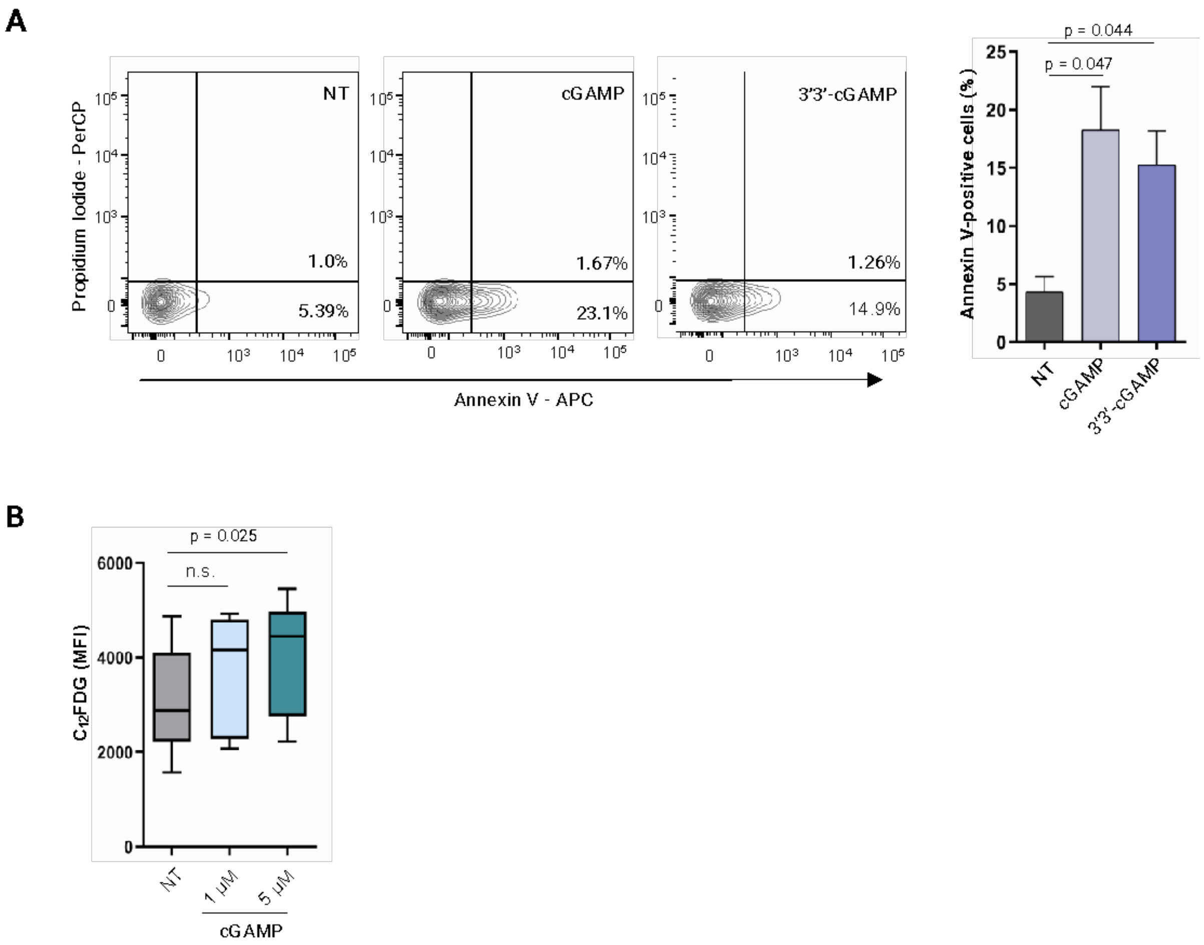

Figure S1- High concentration of exogenous cGAMP and 3'3'-cGAMP triggers apoptosis in SKO-007(J3), Related to Figure 1. A) SKO-007(J3) MM cells were treated with cGAMP or 3'3'-cGAMP 20 μM for 48h; the percentage of apoptotic cells was assessed by flow cytometry using Annexin V – Propidium Iodide double staining. B) SKO-007(J3) MM cells were treated with cGAMP 1 μM or 5 μM for 96h; SA-β Gal activity was measured by flow cytometry using C_{12}FDG. Error bars indicate SEM; statistical significance was calculated using one-way ANOVA.

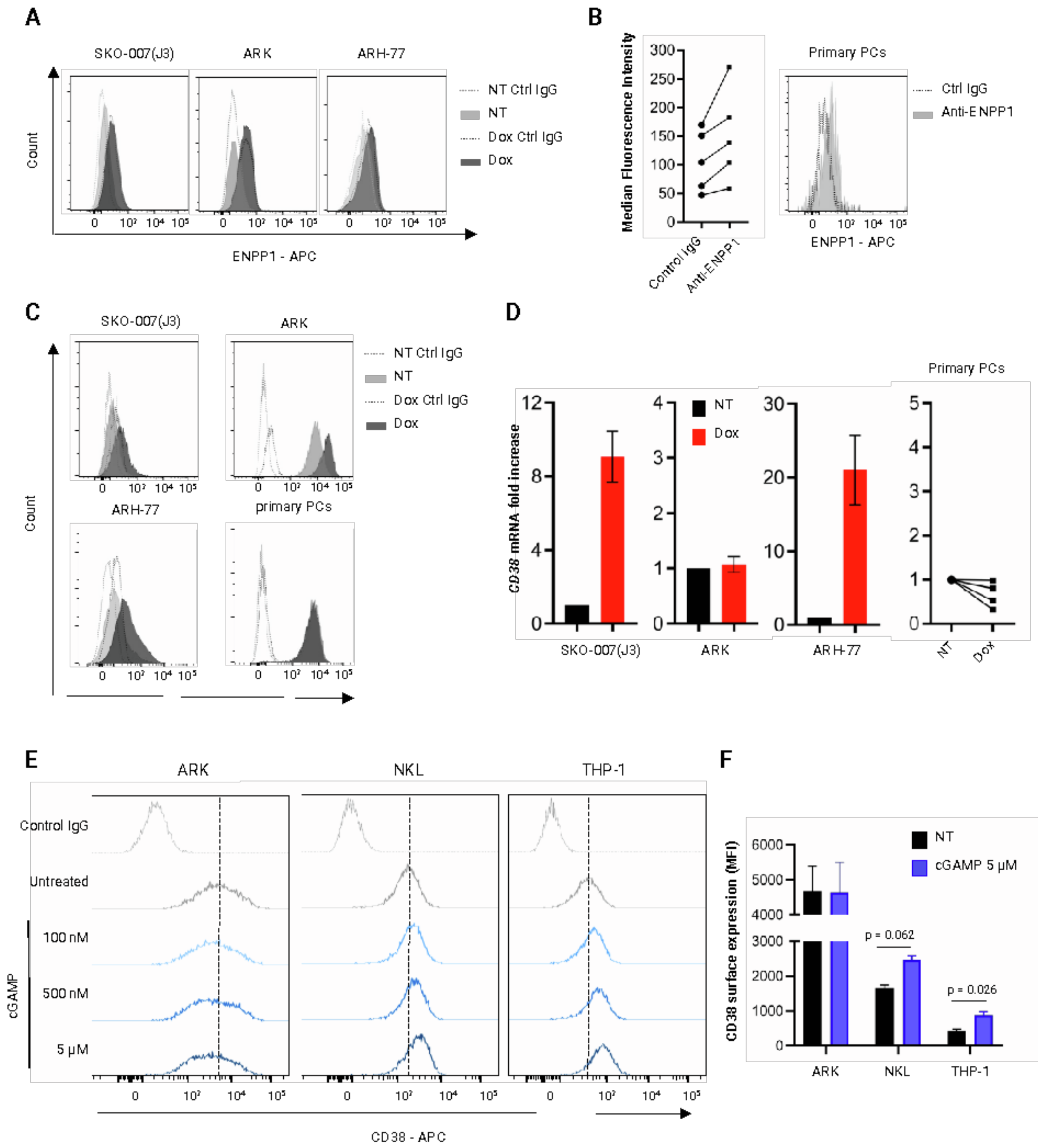
A
SKO-007(J3)
ARK
ARH-77
Count
ENPP1 - APC
NT Ctrl IgG
NT
Dox Ctrl IgG
Dox
B
Median Fluorescence Intensity
Control IgG
Anti-ENPP1
Primary PCs
Ctrl IgG
Anti-ENPP1
ENPP1 - APC
C
SKO-007(J3)
ARK
ARH-77
primary PCs
Count
NT Ctrl IgG
NT
Dox Ctrl IgG
Dox
D
CD38 mRNA fold increase
NT
Dox
SKO-007(J3)
ARK
ARH-77
Primary PCs
E
ARK
NKL
THP-1
Control IgG
Untreated
100 nM
500 nM
5 μM
cGAMP
CD38 - APC
F
CD38 surface expression (MFI)
NT
cGAMP 5 μM
p = 0.062
p = 0.026
ARK
NKL
THP-1

Figure S2- MM cells do not express detectable levels of surface ENPP1, but express CD38, which increases following Dox and cGAMP treatment, Related to Figure 3 and Table 1. A) Surface expression of ENPP1 on untreated and Dox-treated SKO-007(J3), ARK, ARH-77 and B) untreated primary PCs from MM patients (23 to 27 of Table 1) measured by flow cytometry (gated on CD38+CD138+ population). C, D) Surface levels and gene expression of CD38 on untreated and Dox-treated SKO-007(J3), ARK, ARH-77 and primary PCs. E, F) ARK, NKL and THP-1 cells were stimulated with cGAMP (100 nM, 500 nM, 5 μM) for 48h in serum-free medium. CD38 surface levels were then measured by flow cytometry, excluding dead or dying cells. Error bars indicate SEM; statistical significance was calculated using two-tailed paired Student's T test.

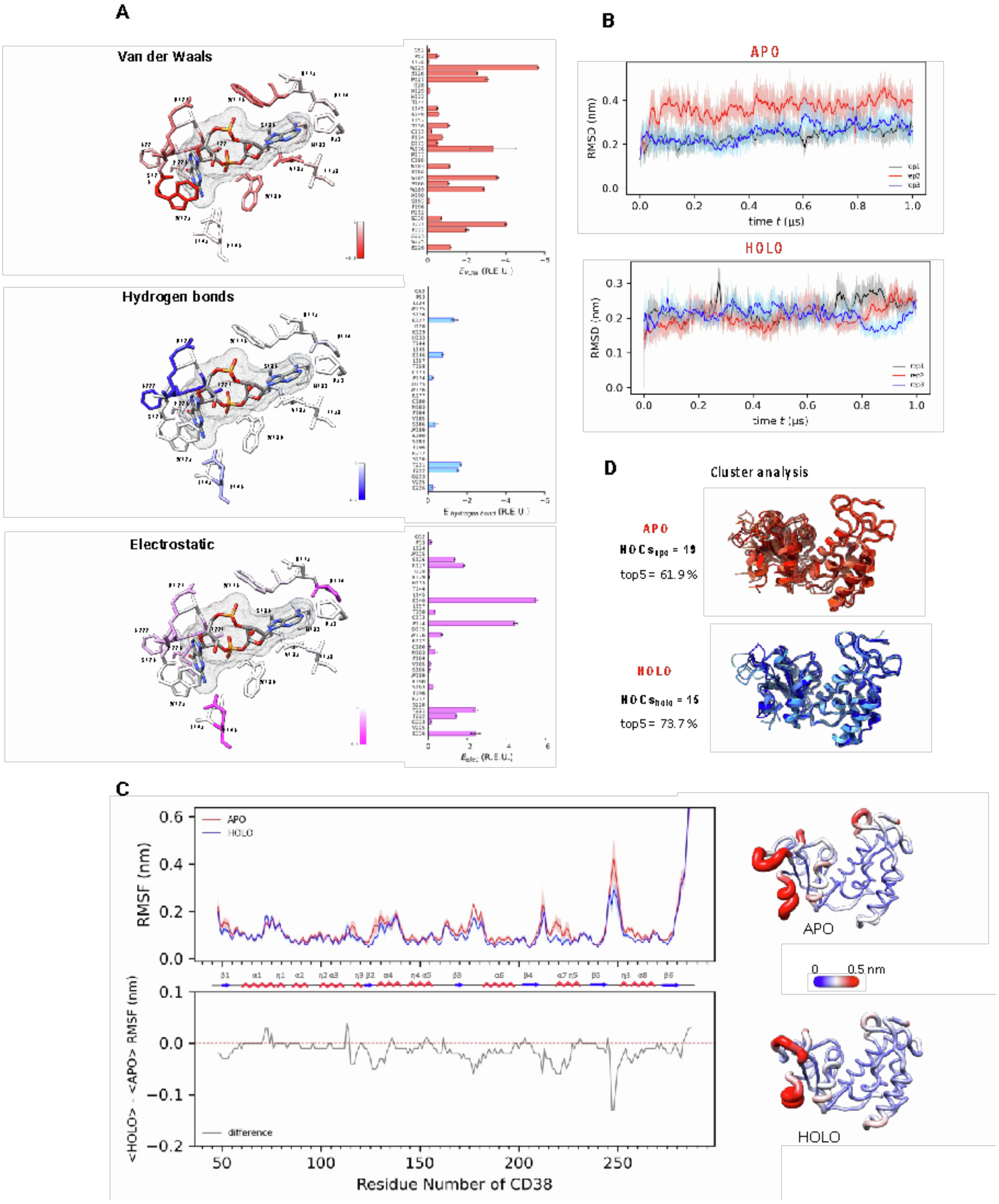
A
Van der Waals
Hydrogen bonds
Electrostatic
B
APO
HOLO
RMSD (nm)
time t (μs)
rep1
rep2
rep3
D
Cluster analysis
APO
HOCs$_{apo}$ = 19
top5 = 61.9 %
HOLO
HOCs$_{holo}$ = 15
top5 = 73.7 %
C
APO
HOLO
RMSF (nm)
<HOLO> - <APO> RMSF (nm)
difference
Residue Number of CD38
0
0.5 nm
APO
HOLO

Figure S3-Molecular docking and MD simulations of CD38-cGAMP complex, Related to Figure 4. A) Predicted CD38-cGAMP complex structure and interactions revealed by molecular docking. Distributions of average VDW, hydrogen bonds and electrostatic interactions between cGAMP (top 10 docking models) and CD38. Energy unit is in Rosetta Energy Unit (R.E.U.). Hydrogen bonds and electrostatic interactions were sparse and specific, while VDW interactions were widely distributed over almost all the residues lining the cGAMP binding pocket. B) Time evolution of unbound (top panel) and *bound* (bottom panel) CD38 backbone RMSD. Bold lines represent running averages, while light lines indicate individual time steps. C) Average Root mean square fluctuation (RMSF) of CD38 unbound (red) and bound (blue) computed for the α-carbons atoms are shown as a function of residue number. Root mean-square fluctuation (RMSF) differences between the bound and unbound CD38. Negative values indicate rigidification of the protein residues upon cGAMP binding (bottom). The averaged Cα positional fluctuation mapped onto the representative unbound and bound structures of CD38 to visualize the largest relative fluctuations. The average fluctuation determined from a combination of all 3 unbound and 3 bound trajectories. The thickness of the cartoon corresponds to the Cα fluctuation. D) RMSD cluster analysis of unbound and bound CD38. The cluster analysis yielded 19 and 15 NOCs (number of clusters) for the unbound and bound respectively, demonstrating less structural heterogeneity in the CD38 bound state. The top five most populated clusters for bound include 73.7% of the total processed frames with respect to 61.9% in unbound: this is a further indication that bound CD38 samples a smaller conformational ensemble compared to the unbound form. cGAMP molecule in the bound state is omitted for clarity.

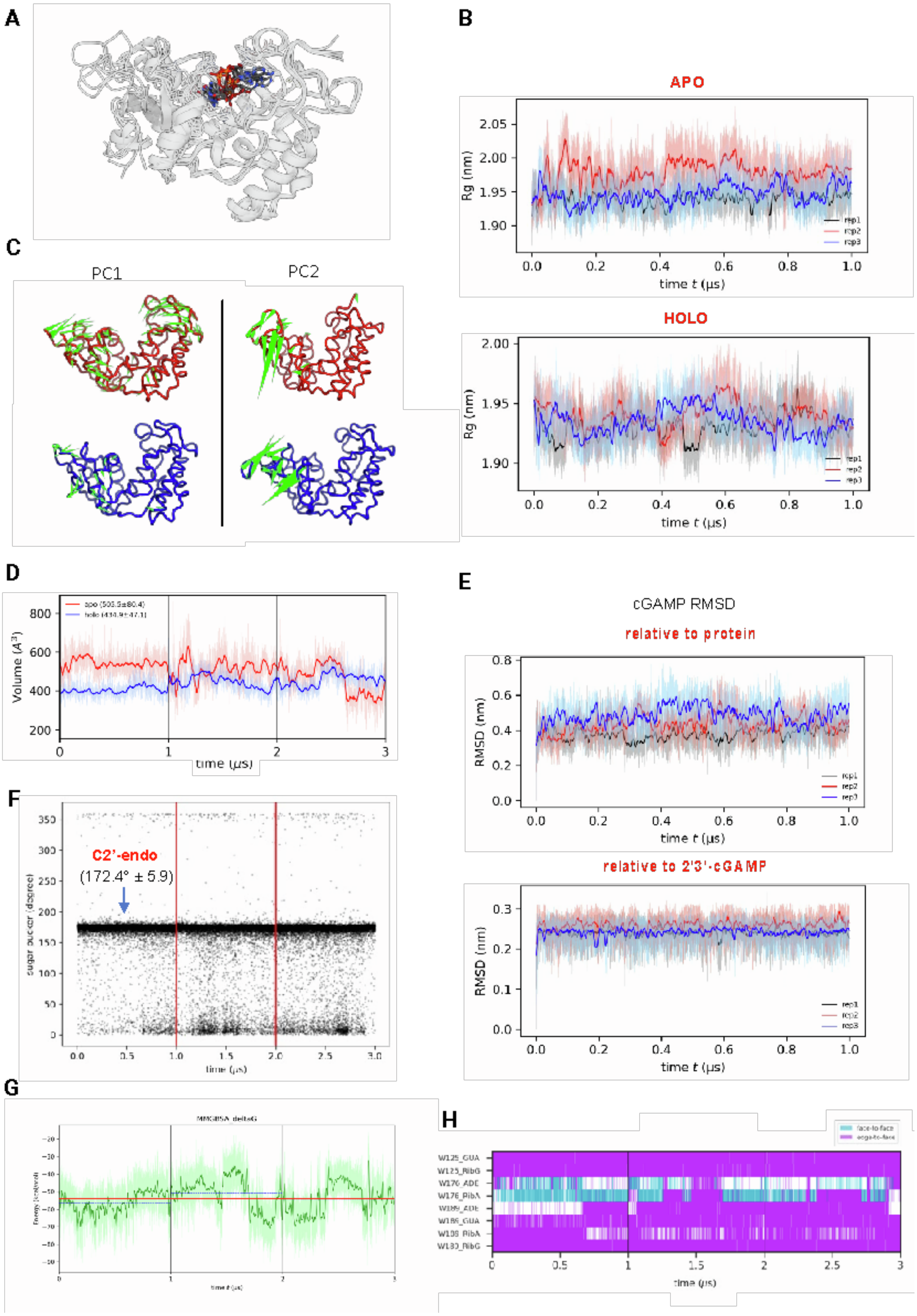
A
B
APO
Rg (nm)
time t (μs)
rep1
rep2
rep3
HOLO
C
PC1
PC2
D
Volume (Å^3)
apo (505.5±80.4)
time (μs)
E
cGAMP RMSD
relative to protein
RMSD (nm)
relative to 2'3'-cGAMP
F
C2'-endo
(172.4° ± 5.9)
sugar pucker (degree)
time (μs)
G
MMGBSA_deltaG
H
face-to-face
W125_GUA
W125_RibG
W176_ADE
W176_RibA
W189_ADE
W189_GUA
W109_RibA
W130_RibG
time (μs)

Figure S4- Structural and energetic features of CD38 in bound and unbound states, Related to Figure 5. A) Structural superposition among representative bound CD38 structures (centroids) extracted from the top 5 populated clusters. cGAMP molecules are depicted as grey sticks. B) Time evolution of unbound (top panel) and bound (bottom panel) CD38 backbone radius of gyration. Bold lines represent running averages, while light lines indicate individual time steps. C) Collective motions corresponding to the first (left) and second (right) PC of unbound and bound CD38. Comparison of the motion described by eigenvector 1 and 2 from the unbound (red) and bound (blue) MD ensembles. Motions are illustrated as linear interpolations between the extreme projections of the structures onto the eigenvectors and are indicated by green arrows. The direction of the arrow in each Cα atom represents the direction of motion, while the length of the arrow characterizes the movement strength. D) Time course of the catalytic pocket volume. Vertical black lines mark delimits three independent 1 μs MD replicas. Bold lines represent running averages, while light lines indicate individual time steps. E) Time evolution of cGAMP heavy atoms with respect to the protein and its binding pocket (top panel) or respect to its starting binding mode (bottom panel). Bold lines represent running averages, while light lines indicate individual time steps. F) Time course of the guanine-linked ribose of cGAMP sugar pucker pseudorotation phase as a function of time from the bound simulation. Vertical red lines mark delimits three independent 1 μs MD replicas. G) Time course of the relative $\Delta G_{binding.}$ Vertical black lines mark delimits three independent 1 μs MD replicas. Dashed blue and red lines indicate the mean of each replica and the total mean computed over all frames, respectively. Bold lines represent running averages, while light lines indicate individual time steps. H) Time evolution of the π-stacking interactions calculated between three selected tryptophan residues (W125, W176, W189) and the adenine (ADE), guanine (GUA), adenine ribose (RibA) and ribose belonging to cGAMP molecule.

Vertical black lines mark delimits three independent 1 μs MD replicas.

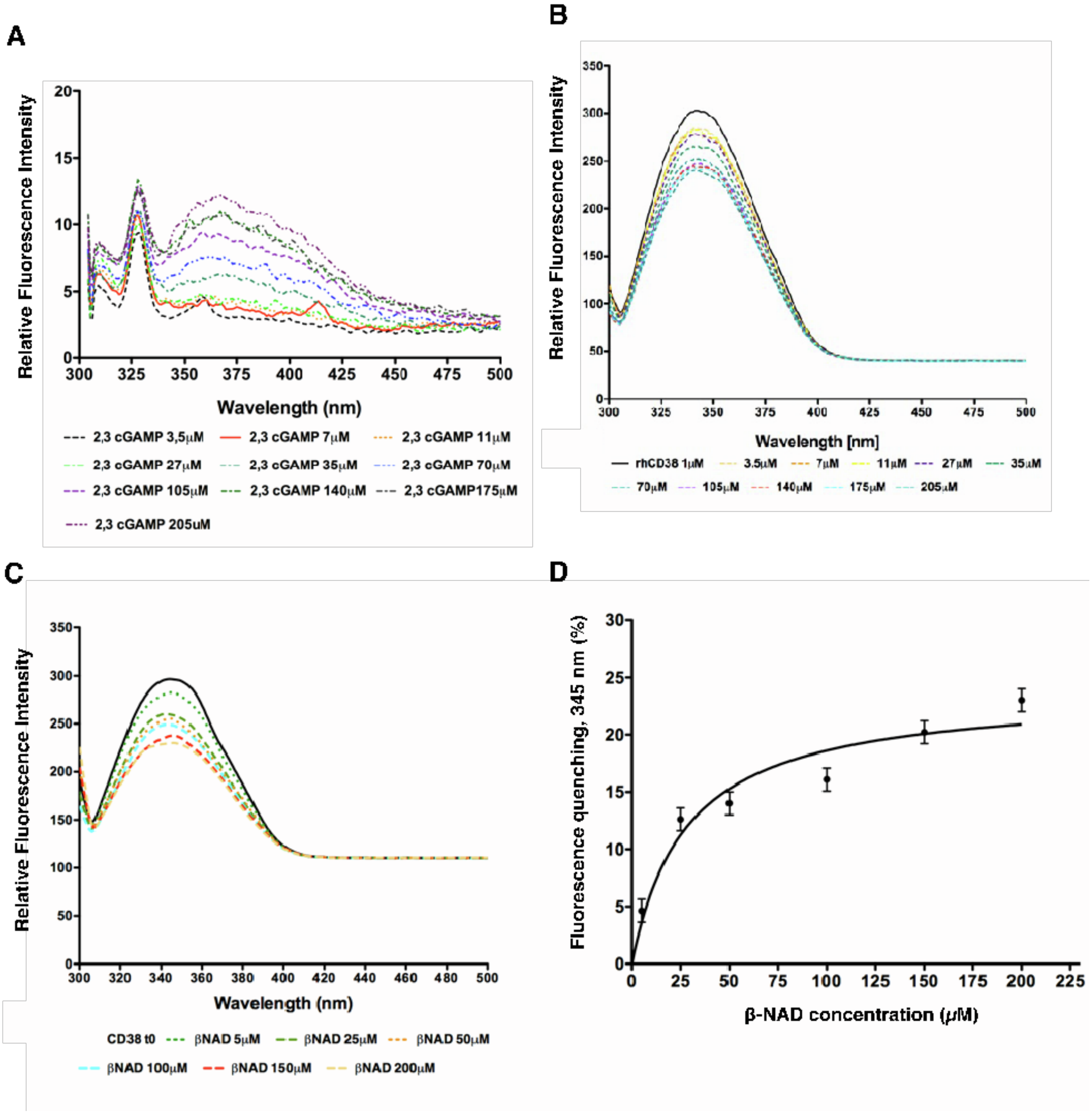

A
Relative Fluorescence Intensity
Wavelength (nm)
2,3 cGAMP 3,5μM
2,3 cGAMP 7μM
2,3 cGAMP 11μM
2,3 cGAMP 27μM
2,3 cGAMP 35μM
2,3 cGAMP 70μM
2,3 cGAMP 105μM
2,3 cGAMP 140μM
2,3 cGAMP175μM
2,3 cGAMP 205uM
B
Relative Fluorescence Intensity
Wavelength [nm]
rhCD38 1μM
3.5μM
7μM
11μM
27μM
35μM
70μM
105μM
140μM
175μM
205μM
C
Relative Fluorescence Intensity
Wavelength (nm)
CD38 t0
βNAD 5μM
βNAD 25μM
βNAD 50μM
βNAD 100μM
βNAD 150μM
βNAD 200μM
D
Fluorescence quenching, 345 nm (%)
β-NAD concentration (μM)

Figure S5- Validation of tryptophan intrinsic fluorescence measurements with cGAMP alone and rhCD38 in presence of 2'3'-cG^{s}A^{s}MP and β-NAD, Related to Figure 6. A) FQ titration of cGAMP (3.5-205 μM) in 50 mM Phosphate buffer pH 7.4 and 50mM NaCl at 25 °C upon excitation at 295 nm. The coloured curves show the fluorescence intensity spectra of cGAMP at different concentrations. B) FQ titration of rhCD38 (1 μM) with increasing 2'3'-cG^{s}A^{s}MP concentrations (0–205 μM) in 50 mM Phosphate buffer pH 7.4 and 50mM NaCl at 25 °C upon excitation at 295 nm. The black curves represent the emission spectrum of rhCD38 in the absence of ligands. The coloured curves show the progressive decrease in fluorescence intensity of rhCD38 at the emission maximum (345 nm) upon the addition of 2'3'-cG^{s}A^{s}MP. C) FQ titration of rhCD38 (1 μM) with increasing β-NAD ligand concentrations (0–200 μM) in 50 mM Phosphate buffer pH 7.4 and 50mM NaCl at 25 °C upon excitation at 295 nm. D) Plot of normalized fluorescence signal at 345 nm expressed as percentage of quenching versus the concentrations of β-NAD. By applying a nonlinear regression fitting, dissociation constants values (K_D) of 28.0 ± 9.4 μM were calculated for the interaction between β-NAD and rhCD38.

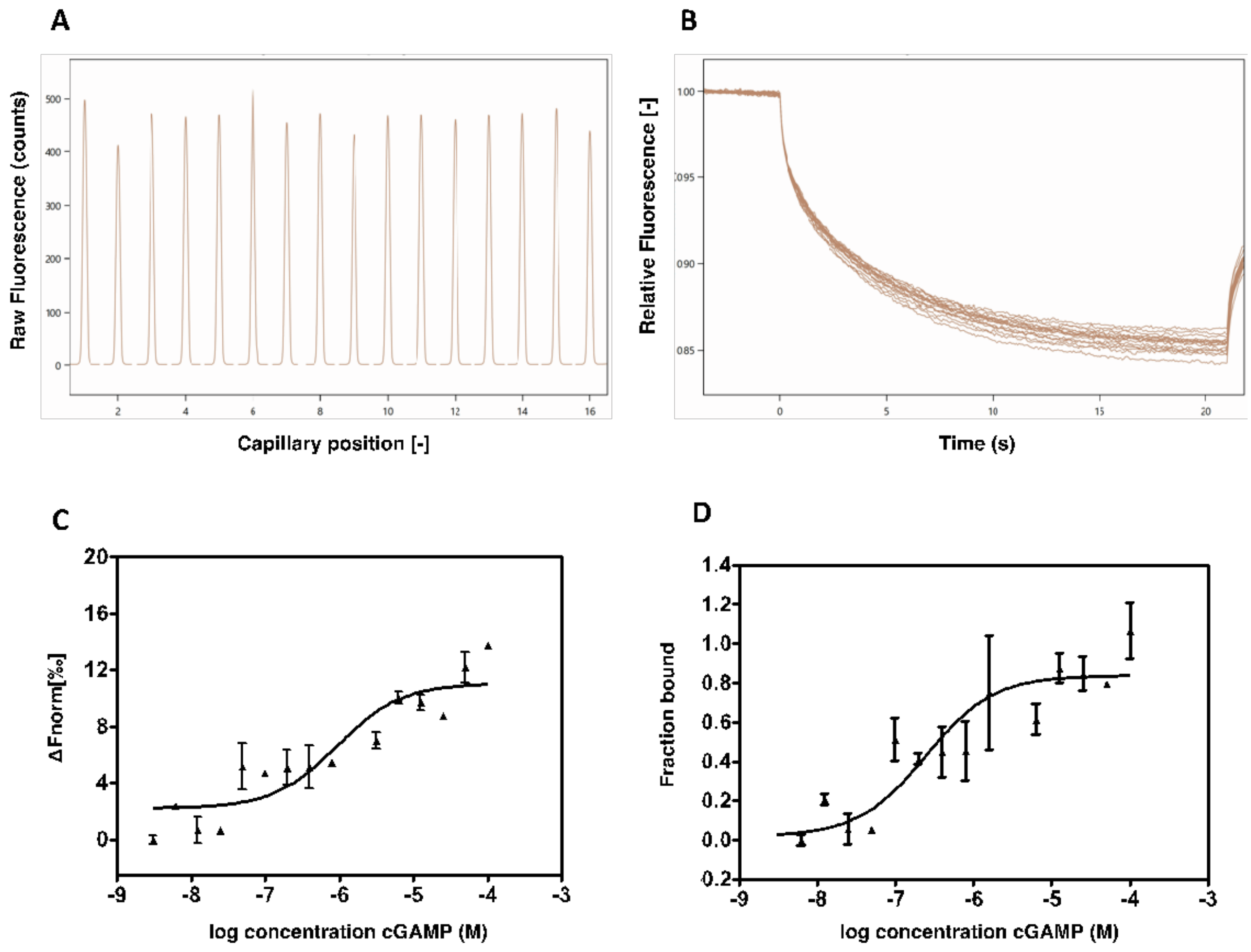

Figure S6- MST interaction analysis of cGAMP with rhENPP1, Related to Figure 6. A) Capillary scan of MST analysis. B, C) MST traces and dose–response curves reported as ΔF_{norm} = normalized fluorescence. Error bars indicate the standard deviation of two independent replicates (N=2). D) MST dose–response curves reported as Fraction Bound. A dissociation constant (K_D ± SD) of 1.1 ± 0.4 µM and signal-to-noise ratios (S/N) of 8.7185 were estimated using the software MO Affinity Analysis, v 2.27.

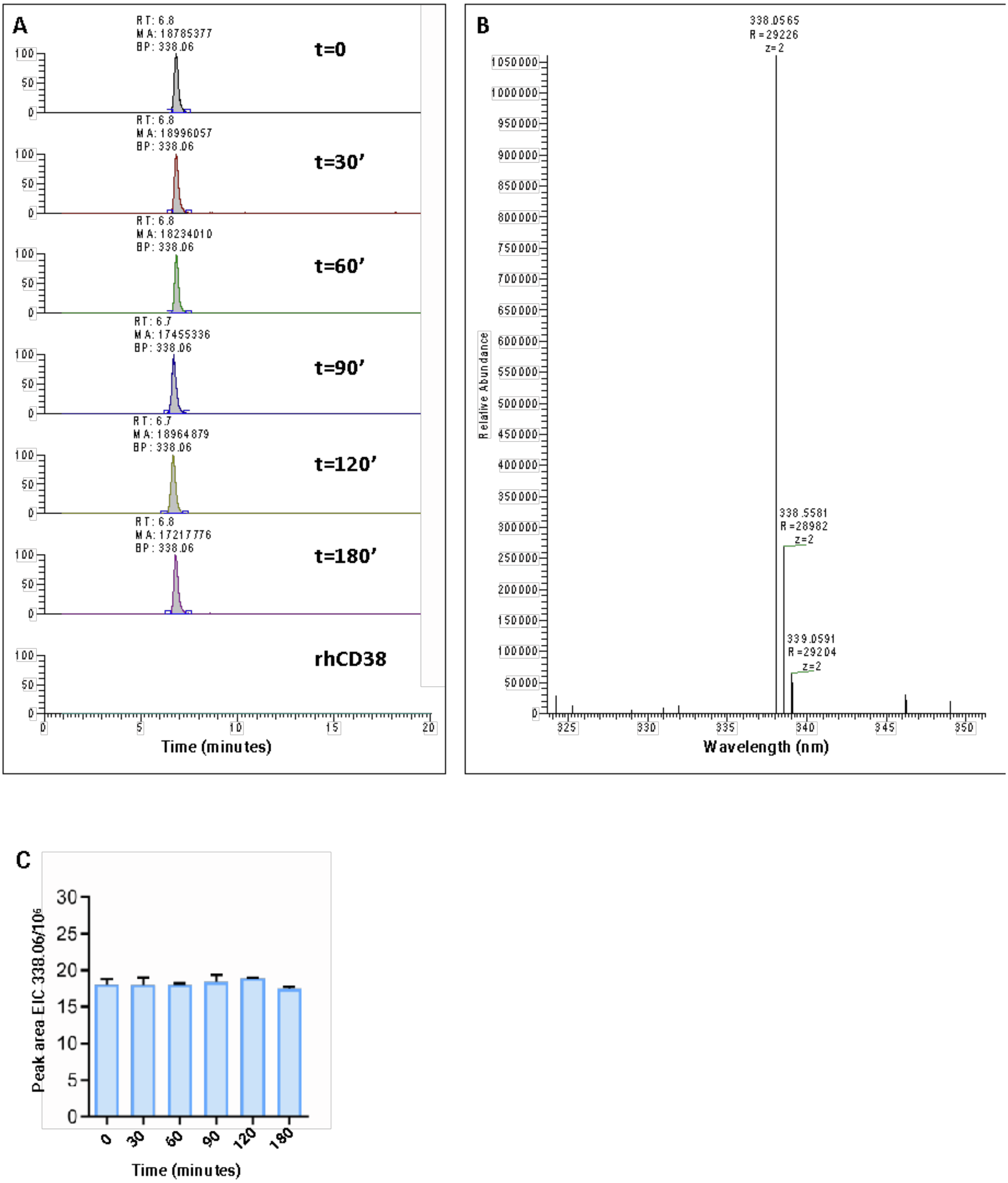
A
RT: 6.8
MA: 18785377
BP: 338.06
t=0
RT: 6.8
MA: 18996057
BP: 338.06
t=30’
RT: 6.8
MA: 18234010
BP: 338.06
t=60’
RT: 6.7
MA: 17455336
BP: 338.06
t=90’
RT: 6.7
MA: 18964879
BP: 338.06
t=120’
RT: 6.8
MA: 17217776
BP: 338.06
t=180’
rhCD38
Time (minutes)
B
338.0565
R=29226
z=2
338.5581
R=28982
z=2
339.0591
R=29204
z=2
Relative Abundance
Wavelength (nm)
C
Peak area EIC 338.06/10⁶
Time (minutes)

Figure S7-LC-MS time-course analyses of cGAMP exposed to rhCD38, Related to Figure 6. A) LC-MS analyses of cGAMP exposed to rhCD38 after 30, 60, 90, 120 and 180 minutes. The dinucleotide was detected as the doubly charged ion at m/z 338.0565. EICs at m/z 338.05-338.7 are reported. Runs are representative of at least duplicate experiments. B) Doubly charged ion of cGAMP detected in all runs. C) Detection of cGAMP at 200 nM in PBS pH 7.0, 37 °C following exposure to rhCD38 at 4 nM at the indicated time points. Values refer to peak areas of Extracted Ion Chromatograms (EIC) obtained monitoring the cGAMP doubly charged ion at m/z 338.060 ± 0.010 ($[M+2H]^{2+}$, experimental m/z 338.0565.

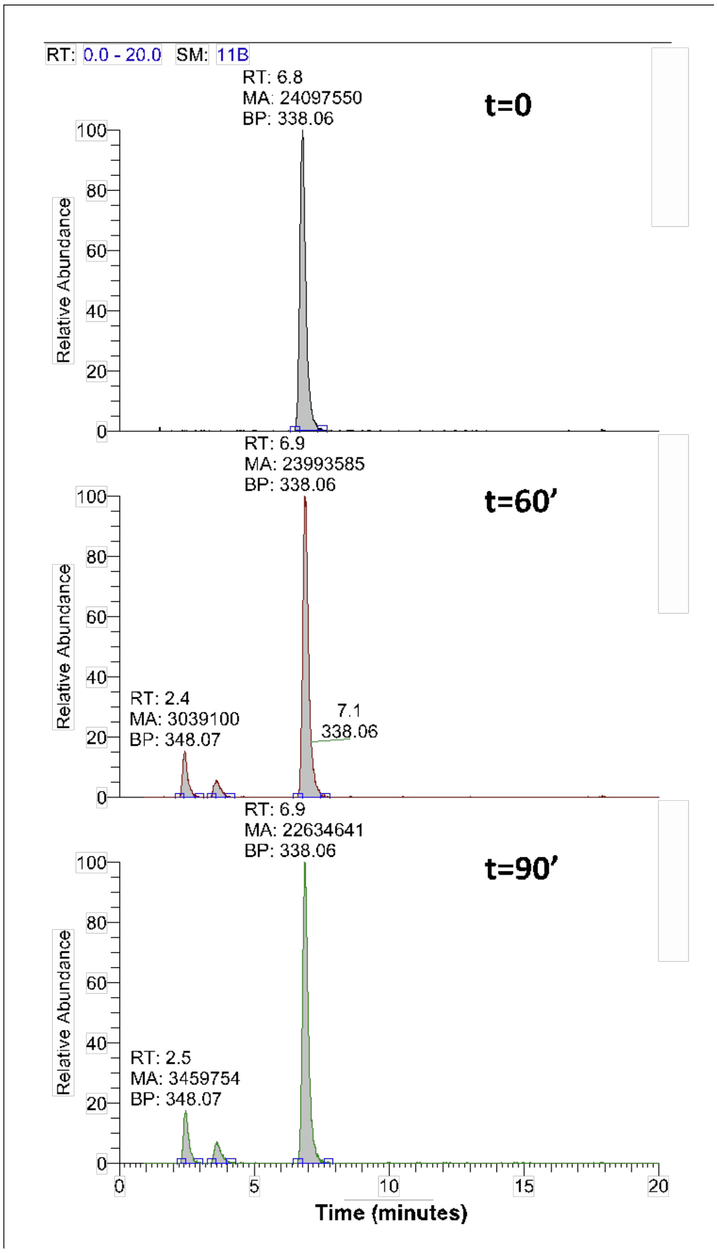

Figure S8-LC-MS time-course analyses of cGAMP exposed to rhENPP1, Related to Figure 6. Hydrolysis of cGAMP (200 nM) by rhENPP1 (4 nM) in 10 mM HEPES pH 7.0. Mass peaks of AMP (2.5 min) and GMP (3.6 min) were detected as extracted ions at m/z 348.07 ± 0.010 and m/z 364.06 ± 0.010.

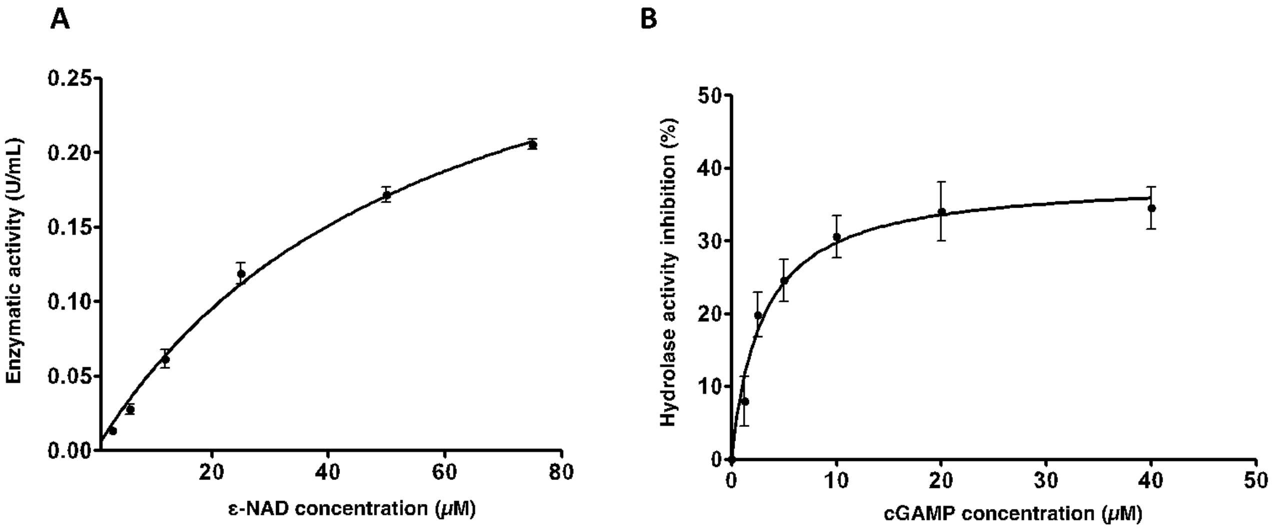

Figure S9- cGAMP inhibits the hydrolase activity of rhCD38, Related to Figure 6. A) Dose-response of CD38 hydrolase activity determined using the ε-NAD substrate at concentrations ranging between 2.5 and 75 μM. Data are reported as enzymatic activity (U/mL) calculated as the ratio between slope and coefficient of molar extinction of enzymes, normalized for the volume of reaction. B) Dose-response inhibition of CD38 hydrolase activity towards ε-NAD (25 μM) by cGAMP at concentrations between 2.5 and 40 μM.

Supplementary tables and their legends

Compound name	Elemental composition	Monoisotopic MW (amu)	$[M+H]^{+ (1)}$/ $[M+2H]^{2+ (2)}$	Experimentally detected m/z	Detected fragments m/z
**cGAMP**	$C_{20}H_{24}N_{10}O_{13}P_2$	674.0999	338.0578(2)	338.0565	136.0616, 152.0564 176.9949, 312.0487 330.0592, 338.8047 392.0142, 476.0348*.
**Linear cGAMP**	$C_{20}H_{26}N_{10}O_{14}P_2$	692.1105	347.0631(2)	347.0627	=
**GMP**	$C_{10}H_{14}N_5O_8P$	363.0580	364.0658(1)	=	=
**AMP**	$C_{10}H_{14}N_5O_7P$	347.0631	348.0709(1)	=	=
**Adenosine**	$C_{10}H_{13}N_5O_4$	267.0967	268.1046(1)	=	=
**Guanosine**	$C_{10}H_{13}N_5O_5$	283.0917	284.0995(1)	=	=
**Adenine**	$C_5H_5N_5$	135.0545	136.0623(1)	=	=
**Guanine**	$C_5H_5N_5O$	151.0494	152.0572(1)	=	=

Table S1. List of compounds sought and identified in LC-MS analysis of cGAMP in the presence of rhCD38, Related to Figure6. cGAMP was mostly detected as doubly charged ion at m/z 338.0565 and fragmented producing the reported daughter ions. A very small mass peak with m/z corresponding to the doubly charged ion of linear cGAMP was also detected (about 3%) in all runs including those in absence of CD38, indicating that it was due to an impurity or spontaneous hydrolysis.

Compound name	Elemental composition	Monoisotopic MW (amu)	$[M+H]^{+ (1)}$/ $[M+2H]^{2+ (2)}$	Experimentally detected m/z
**cGAMP**	$C_{20}H_{24}N_{10}O_{13}P_2$	674.0999	338.0578$^{(2)}$	338.0565
**GMP**	$C_{10}H_{14}N_5O_8P$	363.0580	364.0658$^{(1)}$	364.0656
**AMP**	$C_{10}H_{14}N_5O_7P$	347.0631	348.0708$^{(1)}$	348.0705

Table S2. List of compounds sought and identified in LC-MS analysis of cGAMP in the presence of rhENPP1, Related to Figure 6. cGAMP was mostly detected as a doubly charged ion at m/z 338.0565 and fragmented producing the daughter ions reported in Table S1.
